# SARS-CoV-2 viral titer measurements in Ontario, Canada wastewaters throughout the COVID-19 pandemic

**DOI:** 10.1038/s41597-024-03414-w

**Published:** 2024-06-21

**Authors:** Patrick M. D’Aoust, Nada Hegazy, Nathan T. Ramsay, Minqing Ivy Yang, Hadi A. Dhiyebi, Elizabeth Edwards, Mark R. Servos, Gustavo Ybazeta, Marc Habash, Lawrence Goodridge, Art Poon, Eric Arts, R. Stephen Brown, Sarah Jane Payne, Andrea Kirkwood, Denina Simmons, Jean-Paul Desaulniers, Banu Ormeci, Christopher Kyle, David Bulir, Trevor Charles, R. Michael McKay, Kimberley Gilbride, Claire Oswald, Hui Peng, Vince Pileggi, Menglu L. Wang, Arthur Tong, Diego Orellano, WSI Consortium, Adebowale Adebiyi, Matthew Advani, Simininuoluwa Agboola, Dania Andino, Hussain Aqeel, Yash Badlani, Lena Carolin Bitter, Leslie Bragg, Julia Brasset-Gorny, Patrick Breadner, Stephen Brown, Ronny Chan, Babneet Channa, JinJin Chen, Ryland Corchis-Scott, Matthew Cranney, Hoang Dang, Nora Danna, Rachel Dawe, Christopher DeGroot, Tomas de Melo, Hadi Dhiyebi, Justin Donovan, Walaa Eid, Isaac Ellmen, Joud Abu Farah, Farnaz Farahbakhsh, Meghan Fuzzen, Tim Garant, Qiudi Geng, Ashley Gedge, Alice Gere, Richard Gibson, Kimberly Gilbride, Eyerusalem Goitom, Qinyuan (Crystal) Gong, Tyson Graber, Amanda Hamilton, Blake Haskell, Samina Hayat, Hannifer Ho, Yemurayi Hungwe, Heather Ikert, Golam Islam, Dilan Joseph, Ismail Khan, Richard Kibbee, Jennifer Knapp, James Knockleby, Su-Hyun Kwon, Opeyemi U. Lawal, Line Lomheim, Robert Michael McKay, Ria Menon, Élisabeth Mercier, Zach Miller, Aleksandra M. Mloszewska, Ataollah Mohammadiankia, Shiv Naik, Delaney Nash, Anthony Ng, Abayomi Olabode, Banu Örmeci, Alyssa Overton, Gabriela Jimenez Pabon, Vinthiya Paramananthasivam, Jessica Pardy, Valeria R. Parreira, Lakshmi Pisharody, Samran Prasla, Melinda Precious, Fozia Rizvi, Matthew Santilli, Hooman Sarvi, Mark Servos, Dan Siemon, Carly Sing-Judge, Nivetha Srikanthan, Sean Stephenson, Jianxian (Sunny) Sun, Endang Susilawati, Amir Tehrani, Ocean Thakali, Shen Wan, Martin Wellman, Katie Williams, Ivy Yang, Eli Zeeb, Elizabeth M. Renouf, Christopher T. DeGroot, Robert Delatolla

**Affiliations:** 1 University of Ottawa, Ottawa, ON Canada; 2 University of Toronto, Toronto, ON Canada; 3 University of Waterloo, Waterloo, ON Canada; 4 Health Sciences North Research Institute, Sudbury, ON Canada; 5 University of Guelph, Guelph, ON Canada; 6 Western University, London, ON Canada; 7 Queen’s University, Kingston, ON Canada; 8 Ontario Tech University, Oshawa, ON Canada; 9 Carleton University, Ottawa, ON Canada; 10 Trent University, Peterborough, ON Canada; 11 McMaster University, Hamilton, ON Canada; 12 University of Windsor, Windsor, ON Canada; 13 Toronto Metropolitan University, Toronto, ON Canada; 14 Ontario Ministry of the Environment, Conservation and Parks, Toronto, ON Canada; 15 Children’s Hospital of Eastern Ontario Research Institute, Ottawa, Canada; 16 The Ottawa Hospital Research Institute, Ottawa, Canada

**Keywords:** Epidemiology, Applied microbiology, Civil engineering

## Abstract

During the COVID-19 pandemic, the Province of Ontario, Canada, launched a wastewater surveillance program to monitor SARS-CoV-2, inspired by the early work and successful forecasts of COVID-19 waves in the city of Ottawa, Ontario. This manuscript presents a dataset from January 1, 2021, to March 31, 2023, with RT-qPCR results for SARS-CoV-2 genes and PMMoV from 107 sites across all 34 public health units in Ontario, covering 72% of the province’s and 26.2% of Canada’s population. Sampling occurred 2–7 times weekly, including geographical coordinates, serviced populations, physico-chemical water characteristics, and flowrates. In doing so, this manuscript ensures data availability and metadata preservation to support future research and epidemic preparedness through detailed analyses and modeling. The dataset has been crucial for public health in tracking disease locally, especially with the rise of the Omicron variant and the decline in clinical testing, highlighting wastewater-based surveillance’s role in estimating disease incidence in Ontario.

## Background & Summary

The ongoing COVID-19 pandemic caused by the novel coronavirus disease (SARS-CoV-2) has had a significant global impact on public health, necessitating the swift implementation of innovative disease surveillance and monitoring strategies^1^. Clinical diagnostic testing has been crucial in controlling COVID-19 outbreaks by enabling public health authorities to manage infected individuals through test-trace approaches effectively. While several countries have adopted reverse-transcriptase polymerase chain reaction (RT-PCR) testing and rapid lateral flow antigen self-tests for population-wide monitoring of COVID-19 cases, challenges such as limited tests for asymptomatic individuals and false negatives in antigen tests persist^2^. Furthermore, the development and widespread distribution of mRNA vaccines against symptomatic disease from the SARS-CoV-2 virus marked a significant milestone in mitigating the pandemic’s severity. However, vaccine limitations, including altered efficacy against new variants of concern (VOC)^3–5^, and an unequal global vaccine distribution coupled with the rising costs associated with widespread individual testing at large created a need for sustained, innovative surveillance measures^6–8^. The economic and logistical encumbrances of expansive individual testing, particularly in rural or less developed regions, increased the demand for more cost-effective monitoring strategies. Wastewater-based surveillance (WBS) emerged as a viable complementary tool, addressing the logistical impediments inherent in conventional clinical diagnostic testing.

In recent years, WBS has emerged as a complementary approach for population-wide monitoring of viral diseases, including SARS-CoV-2^9,10^, particularly during periods of lower incidence^11–16^. Since the beginning of the COVID-19 pandemic, it has been demonstrated that individuals infected with SARS-CoV-2, whether symptomatic or not, shed SARS-CoV-2 RNA viral particles in their stools, making wastewater a valuable resource for monitoring the presence and growth of the viral load and disease incidence in a community^17–20^. WBS has further been proven to provide independent and early detection of disease outbreaks, offering insights into the extent of virus spread irrespective of voluntary testing^21–23^. Many countries, including Canada, have adopted WBS as a complementary population-wide monitoring tool for SARS-CoV-2, supporting their public health efforts in controlling the spread of the COVID-19 disease^11,21,24–30^.

Ontario’s WBS program, named the Ontario Wastewater Surveillance Initiative (Ontario WSI), grew from grassroots, academic-led initiatives in the province to identify and measure SARS-CoV-2 in wastewater. The development of analytical methods for SARS-CoV-2 detection in wastewater in the spring of 2020 and the subsequent demonstration of WBS as an early warning system for a COVID-19 outbreak in the City of Ottawa in the summer of 2020 provided a concrete example of the benefits of WBS in the Province of Ontario. This example was presented to a provincial scientific advisory group (the Ontario Science Advisory Table) that was created to inform decision- and policymakers during the pandemic. The Ontario Science Advisory Table proceeded to promote the creation of the WBS program in the province as a complementary surveillance method to current traditional clinical surveillance. These actions led to the development of the Ontario WSI, which was enacted by the Province of Ontario’s government under the leadership of the Ministry of the Environment, Conservation and Parks (MECP)^31^. The program officially began on January 1^st^, 2021, and was developed by leveraging the knowledge, highly trained personnel, and facilities of 13 academic institutions in the province that either had already developed analytical methods for SARS-CoV-2 wastewater analysis, were developing methods of analysis or were willing to adopt methods from other academic institutions and begin analysis. The 13 academic institutions subsequently created the Ontario Wastewater Surveillance Consortium (OWSC), which supports and complements the vision of the Ontario WSI and promotes the advancement of WBS science and research through the sharing and dissemination of acquired data and knowledge.

The Ontario WSI was founded upon a strategic approach that harnesses the advantages of WBS in mapping SARS-CoV-2 prevalence and incidence in communities throughout Ontario, Canada. Situated in east-central Canada, Ontario is the second-largest province in Canada by land area. With a population of approximately 15.5 million residents, it stands as the country’s most populous province (38.8% of Canada’s total population of ~39.9 million residents)^32^. Notably, the province hosts the City of Toronto - the nation’s economic hub, the City of Ottawa - the federal capital, Thunder Bay - home to Canada’s largest urban indigenous population, Leamington - home to Canada’s largest concentration of migrant agri-farm workers, a high degree of connectivity with the United States – five of the monitored sites in the Ontario WSI collectively account for border regions which account for more than 75% of the total cross-border traffic between Canada and the United States. The dataset provided in this manuscript, spanning from January 1^st^, 2021, to March 31^st^, 2023, provides WBS data for 107 major sampling sites, covering 72% of Ontario’s residents. This represents coverage of approximately 26.2% of the total population of Canada (~39.9 million residents). Within the 107 unique sampling sites, 19 were tested by two distinct testing institutions at various times during the program. Consequently, although the dataset in this manuscript reports 126 sites, these represent 107 individual, independent sampling sites. Central to the Ontario WSI’s preliminary objectives is the establishment of a robust data management framework, grounded in FAIR principles (Findable, Accessible, Interoperable, and Reusable)^33^, as these principles have been identified as the utmost importance for SARS-CoV-2 data preservation efforts and studies^27^. As a result, this manuscript has been prepared to ensure continued data availability and metadata preservation, creating an environment appropriate for future research, analysis, modeling, and data synthesis endeavors. The full dissemination of the data is expected to significantly bolster the preparedness of researchers and public health units against future epidemics.

This manuscript presents in detail the methodologies employed by the 13 academic institutions performing the analyses of SARS-CoV-2 in wastewaters throughout the province of Ontario, as well as the dataset containing the quantification of the N1, N2, and/or E SARS-CoV-2 genomic regions. This dataset also includes the quantification of pepper mild mottle virus (PMMoV), as PMMoV has been used as a fecal biomarker and normalizer in the program before further interpretation with public health units. PMMoV normalization has been shown to improve correlations between the SARS-CoV-2 measurements in wastewater and traditional clinical metrics such as reported SARS-CoV-2-associated clinical cases or hospitalizations at some sampling sites by correcting for biases or effects of solid mass flux throughout the sewers on SARS-CoV-2 measurements^11,30,34–36^. It is important to note that PMMoV may not improve correlations of wastewater SARS-CoV-2 measurements to clinical metrics at all sites. The dataset also includes wastewater flowrate measurements and physico-chemical water quality characteristics (pH, total suspended and volatile solids (TSS & VSS), total Kjeldahl nitrogen (TKN), chemical oxygen demand (COD), and total phosphorus) measured at some, but not all, of the wastewater resource recovery facilities (WRRF) sampling sites. As such, if the correlation of wastewater measurements to clinical metrics is an objective of derivative work from the use of the disseminated dataset from this study, then it is important to first determine on a per-site basis if the use of PMMoV, wastewater flowrate, or physico-chemical water quality characteristics as normalizers would further improve correlations with clinical metrics.

## Methods

### Sampling sites, testing institutions, and sample collection

**Fig. 1:**
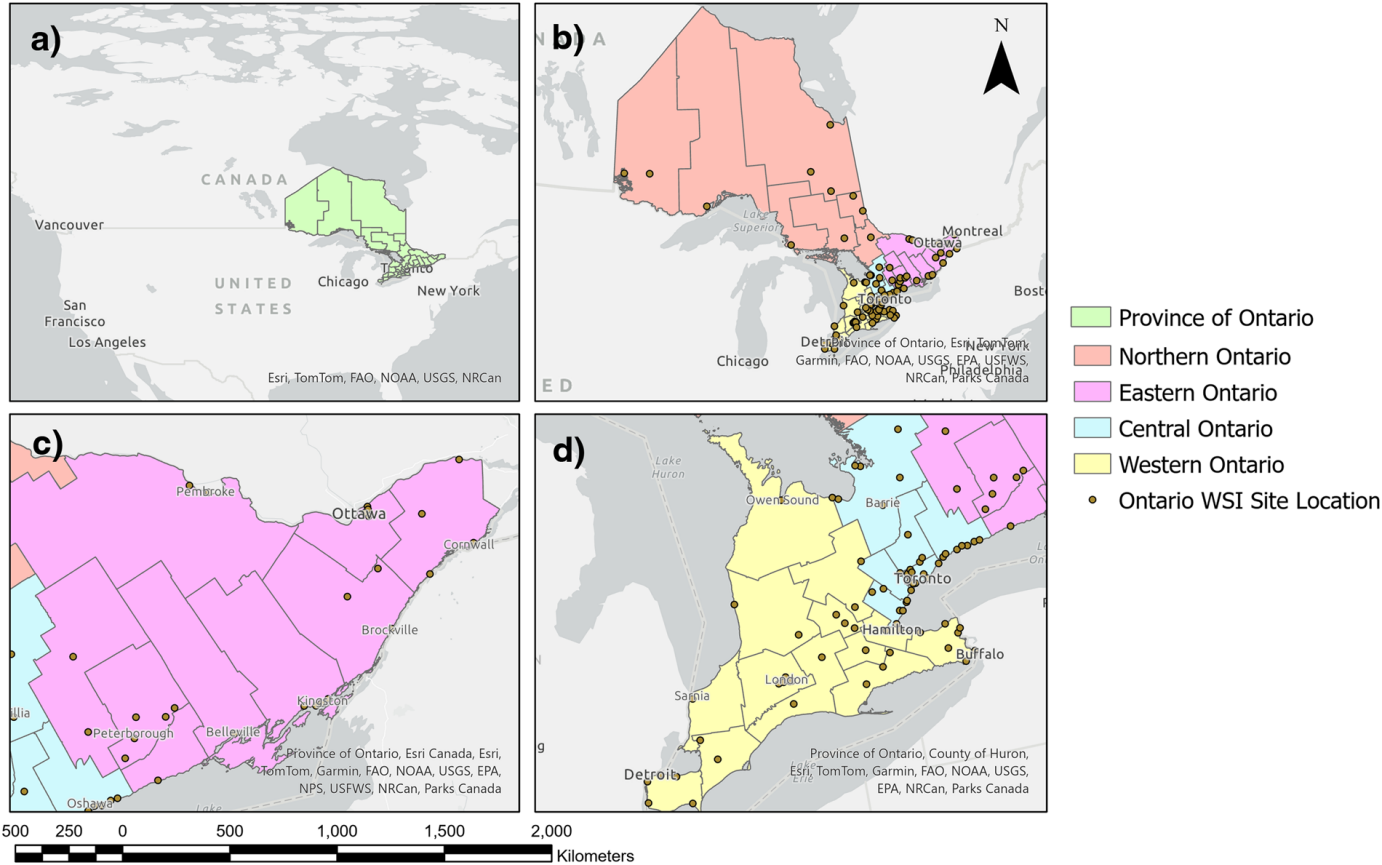
Geographical Overview of Sampling Locations in Ontario, Canada. This panel provides a broader geographical context, illustrating the location of Ontario within North America. (**b**) A comprehensive map of the province of Ontario, with its various public health units distinctly highlighted in various colors, demonstrating the diversity and range of health jurisdictions within the province. Northern Ontario is prominent in this panel, showcasing the public health units in this part of the province. Public health units of Northern Ontario: Thunder Bay District Health Unit, Sudbury and District Health Unit, North Bay Parry Sound District Health Unit, Northwestern Health Unit, Timiskaming Health Unit, Porcupine Health Unit, and Algoma Public Health Unity. (**c**) A detailed view of Eastern Ontario. Public health units of Eastern Ontario: Haliburton, Kawartha, Pine Ridge District Health Unit, Kingston, Frontenac and Lennox and Addington Health Unit, Peterborough Public Health Unit, Ottawa Public Health, Leeds, Grenville and Lanark District Health Unit, Renfrew County and District Health Unit, Hastings and Prince Edward Counties Health Unit. (**d**) A detailed view of Central Ontario and Western Ontario. Public health units of Central Ontario: York Region Public Health, Peel Public Health, Toronto Public Health, Halton Region Health Department, Simcoe Muskoka District Health Unit, and Durham Region Health Department l. Public health units of Western Ontario: Huron Perth Health Unit, Region of Waterloo Public Health, Southwestern Public Health Services, Lambton Public Health, Wellington-Dufferin-Guelph Health Unit, Brant County Health Unit, Middlesex-London Health Unit, Niagara Region Public Health Department, Chatham-Kent Health Unit, Windsor-Essex County Health Unit, Grey Bruce Health Unit, and Haldimand-Norfolk Health Unit.

The sampling of wastewater for the Ontario WSI program was conducted at 107 sampling sites across the province of Ontario by the fourteen testing institutions between January 1^st^, 2021, to March 31^st^, 2023 (Fig. 1a). This program particularly focused on sampling sites at WRRFs located in major population centers, predominantly in Western and Central Ontario, and Eastern Ontario (Fig. 1c,d, respectively). Sampling sites covered 72% of Ontario’s population (approximately 26.2% of Canada’s population)^32^. A close-up view showing areas of the jurisdictions of the 34 provincial public health units (colored geographical areas), along with the sampling sites (brown dots) are shown below in Fig. 1b. A close-up of Eastern Ontario is shown in Fig. 1c,and a close-up view of Central and Western Ontario in Fig. 1d highlights the higher density of sampling sites in these regions.

The list of the 13 testing institutions that collected and analyzed wastewater samples for the Ontario WSI program is as follows: Health Sciences North Research Institute (HSNRI), Carleton University, Queen’s University, Trent University, the University of Ottawa, Ontario Tech University, the Toronto Metropolitan University, the University of Toronto, the University of Guelph, McMaster University, the University of Waterloo, the Western University, and the University of Windsor. The testing institutions have refined the sampling and analysis methodologies throughout the program. On average, samples were collected from all sampling sites between one and seven times a week and were swiftly transported via express courier at 4 °C to the designated laboratory for the duration of the program (January 1^st^, 2021, to March 31^st^, 2023).

**Fig. 2:**
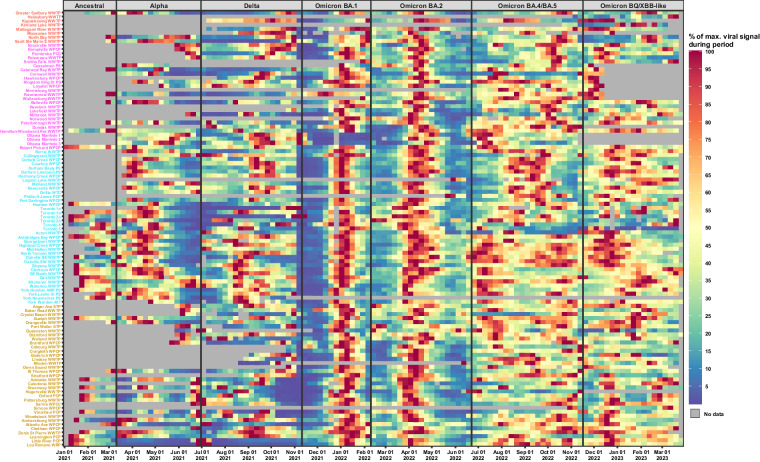
Longitudinal analysis of relative SARS-CoV-2 viral signal (expressed as the % of the max. viral signal) during individual VOC periods across major sampling sites in the Ontario wastewater surveillance initiative (WSI). The graph displays temporal trends in viral signal, expressed as a percentage of the maximum normalized viral signal measured throughout the studied periods (periods are delimited by variant of concern), at various key sampling sites. Color coordination is employed to highlight the magnitude of the PMMoV-normalized viral measurement (as a percentage) compared to the maximum magnitude of the PMMoV-normalized viral measurement during each of the periods, and to highlight peaks of viral signal, per variant of concern (VOC). It is important to note that while notable variations in the data may suggest fluctuations in virus prevalence, these should not be interpreted as direct correlations to distinct outbreaks or pandemic waves without further analysis. Particularly in later VOCs like BA.4/BA.5, and Omicron BQ/XBB-like, the patterns are complex with multiple high signal points. This visualization assists in understanding the spatial and temporal heterogeneity of SARS-CoV-2 prevalence in Ontario. Sites are ordered alphabetically, and site names are colored according to the laboratory which was conducting the testing. Gray sections with lines indicate data unavailability, and the data is resolution is weekly averages.

In the Ontario WSI program, 97.2% of the samples collected were from influent wastewater (untreated wastewater entering the WRRF). Among these, only three sites provided primary sludge wastewater samples (solid waste settled from influent wastewater during the initial treatment stage) (Table S1 in Supplemental Material). Influent samples, typically gathered before any screening or grit removal processes (which eliminates large material, debris, and large inorganic particles) or from facilities lacking this process, were collected from 67 sites and are labeled as “Influent” in Table S1. Post-grit influent samples (taken after screening of large material and debris and after the removal of large inorganic particles) were collected from 33 sites, while post-screen influent samples (acquired after removal of large material and debris only) were collected from four sites. Within the Ontario WSI program, most sampling sites (100 out of the 107) employed autosamplers to gather 24-hour composite samples, typically collected three to seven times a week during each sampling interval. Seven sampling sites were unable to install or utilize autosamplers; of these, five sites collected 24-hour composite grab samples, and the remaining two sites used passive sampling methods (Table S1). Table S1 below provides comprehensive details regarding the testing institution, the sampling site, the type of wastewater sample, the sampling method, the population in the vicinity of the WRRF/upstream sampling site, and the average flowrate of the WRRF/upstream sampling site (including minimum and maximum flows, when available and validated by the responsible municipality) for each sampling site. The names of the testing institutions in this table are color-coordinated with the corresponding testing sites shown in the heatmap of Fig. 2.

### Sample analysis: RNA enrichment and extraction

The harvested wastewater samples from all sampling sites under the Ontario WSI program underwent transportation on ice by couriers and underwent SARS-CoV-2 RNA enrichment and extraction within 48–72 hours of sample arrival. While sample enrichment and nucleic acid extraction methods exhibited minor variations across testing institutions, each testing institution showcased proficiency and general equivalence, as evidenced by recurrent interlaboratory tests throughout the province (data not shown)^37,38^. These tests were administered by an MECP-led inter-laboratory proficiency testing program. Early partitioning experiments during the pandemic suggested that methodologies emphasizing the enrichment of the wastewater samples’ solid fraction produced more consistent and robust viral signals during the low incidence of COVID-19, which may have directed the method selection of the testing institution. Table S2 in Supplemental Material presents comprehensive analytical method information from each testing institution, including specific sample RNA enrichment methods, nucleic acid extraction methods, and any noted deviations from the manufacturer’s guidelines. Following extraction, nucleic acids were stored at 4 °C for up to 24 hours, −20 °C for 24–48 hours, or −80 °C for 24–72 hours (depending on the laboratory’s internal procedures and expected extra hold time), and typically analyzed within 72 hours using RT-qPCR. Subsequently, the surplus extracts were stored at −80 °C.

### Sample analysis: RT-qPCR

Testing institutions participating in the Ontario WSI program adhered to specific RT-qPCR analysis guidelines^39,40^, where every sample was required to be analyzed for two gene region targets of the SARS-CoV-2 genome, the N1, N2, N200, and/or E genomic regions. Additionally, each testing institution was required to measure PMMoV in each sample, serving as an endogenous fecal biomarker used to normalize the wastewater surveillance data. The methodologies for RT-qPCR, inclusive of supermix and targets, displayed minor variations across testing institutions. Table S2 provides detailed information about each laboratory’s RT-qPCR methodology, including the PCR instrument type, supermix, targeted SARS-CoV-2 gene regions, and the RT-qPCR reaction conditions for both SARS-CoV-2 and PMMoV targets. Moreover, Table S3 in Supplemental Material presents the detailed method and RT-qPCR controls, sensitivity (assay limit of detection; ‘ALOD’ and quantification; ‘ALOQ’, if available), and quality controls implemented by the testing institutions participating in the Ontario WSI program.

### Data qualifiers and quality controls

The ALOD and ALOQ values for each testing institution were determined as previously described^11^. In brief – The ALOD of the RT-qPCR and RT-ddPCR assays for SARS-CoV-2 and PMMoV targets were calculated by determining the number of genomic copies/reactions which corresponded to an average positive detection rate greater or equal to 95%, as per recommendations from the MIQE guidelines^41^. The ALOQ of the RT-qPCR and RT-ddPCR assays for SARS-CoV-2 and PMMoV targets were determined by calculating the coefficient of variation of various concentrations of the analytes and mathematically determining the measurement when the coefficient of variation was equal to 35% (CV35%). Inhibition checks were employed by every testing institution to verify if inhibition was present during the analysis of the wastewater samples. Some testing institutions performed additional sensitivity checks using spike-ins or RNA measurements. Key QA/QC criteria such as calibration curve linearity and PCR efficiency data were also specified by each testing institution. In the dataset, data generated from each testing institution’s laboratory was labeled with the following quality flags if necessary: ‘ND’ to indicate a non-detect/zero value, ‘J’ for values near the lowest point of the standard curve (between the LOQ and the Y-intercept of the standard curve), and ‘UJ’ for trace values where amplification of signal occurred beyond the Y-intercept of the experiment-specific standard curve, (typically below 1 copy/reaction, but still amplified). Table S3 provides detailed information regarding the assay limits of detection and quantification, inhibition checks, sensitivity checks, quality assurance, and quality control checks employed by each testing institution.

### Normalization

The dataset provided on GitHub (please refer to Section 3 for further details) presents measurements of SARS-CoV-2 viral N1, N2, N200, and/or E genomic copies in wastewater respective to each sampling site. Additionally, it contains data on PMMoV viral copies. The Ontario WSI program utilized the PMMoV measurement as a standardizing factor, normalizing the WBS data by calculating the ratio of SARS-CoV-2 copies to PMMoV copies before conducting further analysis. This normalization approach, using PMMoV as a reference, was adopted by several public health units to understand COVID-19 incidence and integrate the WBS data with conventional surveillance data, such as clinical data or hospitalizations. Notably, wastewater flowrate was not used as a normalizing factor for WBS data or any public health units. The users of this dataset need to note that they need to independently apply normalization using PMMoV measurements or flowrates to the SARS-CoV-2 measurements if they require normalized WBS data for their analyses.

### Data visualization

Figure 2 showcases a heatmap generated using R programming language and ggplot2 (version 3.4.2), illustrating the prevalence of various SARS-CoV-2 variants of concern (VOC) between January 1^st^, 2021, and March 31^st^, 2023, in Ontario’s wastewater samples. The Province of Ontario has been actively monitoring SARS-CoV-2 variants of concern (VOC) using whole genome sequencing (WGS) of select wastewater samples collected from the WSI program since February 3^rd^, 2021^42^. Between January 1^st^, 2021, and March 31^st^, 2021, Ontario identified the following VOCs in its 34 public health units: ancestral/wildtype, Alpha (B.1.1.7), Delta (B.1.617.2), and multiple Omicron sublineages including Omicron BA.1 (B,1.1.516.1), Omicron BA.2 (B.1.1.516.2), Omicron BA.4/BA.5-like, and Omicron BQ/XBB-like sublineages^42,43^. However, the detection of these VOCs was not uniform across all Ontario WSI sampling sites. For visualization purposes, the heatmap’s periods are approximated to 20–40 days around each major VOC wave, using the dataset from the Ontario WSI program. The y-axis of the heatmap lists the sampling sites in alphabetical order, with color-coordination indicating the testing institutions that analyzed the samples (please refer to Tables 1–3 for testing institutions’ details).

Figure 2 employs PMMoV-normalized SARS-CoV-2, ‘copies per copies’ (cpcp) data, which is transformed into a ‘percent of maximum viral signal’ (pmvs) metric. This transformation is necessary, as plotting the raw PMMoV-normalized SARS-CoV-2 data could obscure the data from sites with lower viral loads due to variations in normalized viral load across different sites. The process involves calculating the weekly average copies per copy for each sampling site and subsequently segregating the data by VOC type. For each VOC, the pmvs metric is determined by identifying its maximum weekly average copies per copy and dividing each corresponding data point by its identified maximum. Thus, each data point represents a weekly average percentage of the highest viral signal observed during the dominant phase of the respective VOC, emphasizing the peaks in viral signals during the various VOC waves and providing a nuanced perspective on the geographical and temporal spread of VOCs across Ontario. It is noteworthy that the grey bars on the heatmap denote periods without data, corresponding to times before wastewater analysis commenced at specific Ontario WSI sites (as detailed in Table S1).

## Data Records

The complete dataset presented in this manuscript is fully available for download in.CSV format from Zenodo^44^ and our GitHub repository (https://github.com/OntarioWastewaterSurveillanceConsortium/sars-cov-2-data). These datasets encompass gene quantifications (SARS-CoV-2 N1, N2, N200, and/or E, and PMMoV) in copies/L, collected between (as early as) January 1^st^, 2021 and March 31^st^, 2023, from 107 major sampling sites distributed across the province of Ontario. The datasets are systematically organized into testing institution-specific folders. Each folder contains the following.CSV files: **AssayMethod.CSV** – a short description of the assay methods used throughout the studied period included in this manuscript; **Instrument.CSV** – a short description of the instrument used for RT-qPCR, dPCR or ddPCR analysis; **Lab.CSV** – a short description of the laboratory/testing institution which performed the testing; **Reporter.CSV** – a short description/list of the individuals who performed the data validation and entry; **Sample.CSV** – a comprehensive record of the sample type, sample collection point, and volume of sample collected; **Site.CSV** – a short description of the name and type of sampling sites along with their respective geographical coordinates; **SiteMeasure.CSV** – a short description of operational measurements (i.e., single variable) obtained at the wastewater sampling site, and includes data that is commonly collected by staff at wastewater treatment facilities and sites; these measures that are not performed on the wastewater sample but provide additional context for necessary interpretation of the results (for example: flow); **WWMeasure.CSV** – testing institution analysis results (RT-qPCR or physico-chemical analysis) (i.e., single variable) from wastewater samples; includes data that is commonly collected by staff at wastewater testing institution where the measurement is performed using an assay method (see AssayMethod.CSV), but can also be performed using specific instruments (see Instruments.CSV). The attributes and a short description for each CSV file hosted on GitHub are listed below:

**AssayMethod.CSV**

- **assayMethodID:** (Primary key) Unique identifier for the assay method.
- **instrumentID:** (Foreign Key) Links with the Instrument table to describe instruments used for the measurement.
- **name:** Name of the assay method.
- **version:** Version of the assay. Semantic versioning is recommended.
- **summary:** Short description of the assay and how it is different from the other assay methods.
- **referenceLink:** Link to standard operating procedure.
- **date:** Date on which the assayMethod was created or updated (for version update).
- **sampleSizeL**: Size of the sample that is analyzed in liters.
- **inhibition:** Description of inhibition control is to be provided noting that inhibitory substances may be present that impede or prevent PCR from running efficiently or effectively, ultimately resulting in delayed Cq quantification (higher Cq) for the actual target of the analysis.
- **surrogateRecovery:** This is the proportion of a specific target within an analytical portion of a test material (e.g., wastewater sample) that was successfully extracted and detected/represented by the final measurement (see Protocol for additional details)
- **mCF:** Concentration Factor (CF) is the degree to which the concentration observed in the final qPCR assay volume has been magnified compared to concentration of the analyte in the original sample (see Box 2.1 Protocol^37^).
- **mESV:** Effective Sample Size/Volume (ESV) is the amount of the original sample size/volume that was actually analyzed in a qPCR reaction (see Box 2.1 Protocol)
- **sloq:** Sample limit of quantification (SLOQ) is defined as “the concentration of a target that can be quantified with an acceptable level of precision when present in a sample” (Ahmed *et al*.^20^).
- **slod:** Sample limit of detection (SLOD) is the minimum level of a target within a sample that would be consistently detectable considering all sample processing steps, from sample concentration leading up to and including amplification and quantification steps of RT-qPCR.
- **unit: Unit used by this method and applicable to the LOD and LOQ**.
  - **gcPMMoV:** Gene copies per copy of PMMoV.
  - **gcMl:** Gene copies per milliliter.
  - **gcGms:** Gene copies per gram solids.
  - **gcL:** Gene copies per liter.
  - **gcCrA:** Gene copies per copy of crAssphage.
  - **other:** Other measurement of viral copies. Add description to unitOther.
  - **unitOther:** Unit used by this method, and that are applicable to the LOD and LOQ.

**Instrument.CSV**

- **instrumentID:** (Primary key) Unique identifier of instrument used for Assay Method.
- **name:** Name of the instrument used to perform the measurement.
- **model:** Model number or version of the instrument.
- **description:** Description of the instrument.
- **referenceLink:** Link to reference for the instrument.
- **type: Type of instrument used to perform the measurement**.
  - **online:** An online sensor
  - **lab:** Offline laboratory analysis
  - **hand:** A handheld measurement analyzer.
  - **atline:** An atline analyzer with sampler.
  - **other:** Another type of measurement instrument. Add description to Instrument typeOther.
  - **typeOther:** Description of the instrument in case it is not listed in Instrument type.

**Lab.CSV**

- **labID:** (Primary key) Unique identifier for the laboratory.
- **name:** Name corresponding to lab.
- **contactName:** Contact person or group, for the lab.
- **contactEmail:** Contact e-mail address, for the lab.
- **contactPhone:** Contact phone number, for the lab.
- **contactPhoneExt:** Contact phone number extension, for the lab.
- **updateDate:** Date information was provided or updated.

**Reporter.CSV**

- **reporterID:** (Primary Key) Unique identifier for the person or organization that is reporting the data.
- **contactName:** Full Name of the reporter, either an organization or individual.
- **contactEmail:** Contact e-mail address.
- **contactPhone:** Contact phone number.
- **contactPhoneExt:** Contact phone number extension, for reporter.
- **notes:** Any additional notes.

**Sample.CSV**

- **sampleID:** (Primary Key) Unique identification for sample. Suggestion: siteID-date-index.
- **siteID:** (Foreign key) Links with the Site table to describe the site of sampling.
- **type:** Type of sample.
- **collection:** Method used to collect the data.
- **dateTime:** For grab samples this is the date, time and time zone the sample was taken.
- **dateTimeStart:** For integrated time averaged samples this is the date, time and time zone the sample was started being taken.
- **dateTimeEnd:** For integrated time average samples this is the date, time and time zone the sample was finished being taken.
- **sizeL:** Total field sample volume, prior to sub-sampling, of wastewater or sludge sampled.
- **fieldSampleTempC:** Temperature that the sample is stored at while it is being sampled. This field is mainly relevant for composite samples which are either kept at ambient temperature or refrigerated while being sampled.
- **shippedOnIce:** Was the sample kept cool while being shipped to the lab?
- **storageTempC:** Temperature that the sample is stored in Celsius.
- **sentDate:** The date the sample was sent from the site to the laboratory for analysis.
- **recDate:** The date the sample was received by the laboratory for analysis.
- **notes:** Any additional notes
- **type: The types of samples that can be collected are as follows:**
  - **pSludge:** Sludge produced by primary clarifiers.
  - **rawWW:** Raw wastewater.
  - **pstGrit:** Raw wastewater after a treatment plant’s headworks.

- **The sample collection methodologies are as follows:**
  - **cp:** Composite sample.
  - **cpgrb:** Grab composite sample.
  - **cpTP24h:** A time proportional 24-hour composite sample generally collected by an autosampler.
  - **cpFP24h:** A flow proportional 24-hour composite sample generally collected by an autosampler.
  - **grb:** A single large representative grab sample.
  - **grbCp8h:** An 8-hour composite with 8 grab samples each taken once per hour, generally manually performed.
  - **grbCp3h:** A 3-hour composite with 3 grab samples each taken once per hour, generally manually performed.

- **grbCp3:** A grab-composite sample composed of 3 separate grab samples.
  - **mooreSw:** Moore swab passive sample.

**Site.CSV**

- **siteID:** (Primary Key) Unique identifier for the site where wastewater sample was taken.
- **name:** Given name to the site. Site name could be a treatment plant, campus, institution, or sewer site, etc.
- **description:** Description of wastewater site (city, building, street, etc.) to better identify the site of the sampling point.
- **type: Type of site or institution where sample was taken**.
  - **airPln:** Airplane.
  - **corFcil:** Correctional facility.
  - **school:** School.
  - **hosptl**: Hospital.
  - **ltcf:** Long-term care facility.
  - **swgTrck:** Sewage truck.
  - **uCampus:** University campus.
  - **mSwrPpl:** Major sewer pipeline.
  - **pStat:** Pumping station.
  - **holdTnk:** Holding tank.
  - **retPond:** Retention pond.
  - **wwtpMuC:** Municipal wastewater treatment plant for combined sewage.
  - **wwtpMuS:** Municipal wastewater treatment plant for sanitary sewage only.
  - **wwtpInd:** Industrial wastewater treatment plant.
  - **lagoon:** Lagoon system for municipal wastewater treatment.
  - **septTnk:** Septic tank.
  - **other:** Other site type. Add description to typeOther.
  - **typeOther:** Description of the site when the site is not listed. See siteType.

- **healthRegion:** Local Health Integration Network (LHIN). See www.lhins.on.ca to locate a LHIN.
- **publicHealthDepartment:** Public Health Unit.
- **geoLat:** Site geographical location, latitude in decimal coordinates, i.e.: (45.424721)
- **geoLong:** Site geographical location, longitude in decimal coordinates, i.e.: (-75.695000)
- **notes:** Any additional notes.

**SiteMeasure.CSV**

- **uSiteMeasureID:** (Primary Key) Unique identifier for each measurement for a site.
- **siteID:** (Foreign Key) Links with the Site table to describe the site of measurement.
- **sampleID:** (Foreign Key) Links with the Sample table to describe the sample.
- **reporterID:** (Foreign key) Links with the reporter that is responsible for the data.
- **dateTime:** The date and time the measurement was performed.
- **type: The type of measurement that was performed. The prefix ‘env’ is used for environmental variables, whereas ‘ww’ indicates a measurement on wastewater or primary sludge**.
  - **envTemp:** Environmental temperature.
  - **envRnF:** Rain fall (i.e., amount of precipitation in the form of rain).
  - **envSnwF:** Snow fall (i.e., amount of precipitation in the form of snow).
  - **envSnwD:** Total depth of snow on the ground.
  - **wwFlow:** Flow of wastewater.
  - **wwTemp:** Temperature of the wastewater.
  - **wwTSS:** Total suspended solids concentration of the wastewater.
  - **wwCOD:** Chemical oxygen demand of the wastewater.
  - **wwTurb:** Turbidity of the wastewater.
  - **wwOPhos:** Ortho-phosphate concentration.
  - **wwNH4N:** Ammonium nitrogen concentration, as N.
  - **wwTN:** Total nitrogen concentration, as N.
  - **wwpH:** pH of the wastewater.
  - **wwCond**: Conductivity of the wastewater.
  - **other:** Any other type of measurement. Add description to typeOther.

- **typeOther:** Description of the measurement in case it is not listed in type.
- **typeDescription:** Additional information on the performed measurement.
- **aggregation: When reporting an aggregate measurement, this field describes the method used**.
  - **single:** This value is not an aggregate measurement in any way (i.e., not a mean, median, max or any other) and can be a replicate value.
  - **mean:** Arithmetic mean.
  - **meanNr:** Arithmetic mean, normalized.
  - **geoMn:** Geometric mean
  - **geoMnNr:** Geometric mean, normalized.
  - **median:** Median.
  - **min:** Lowest value in a range of values.
  - **max:** Highest value in a range of values.
  - **sd:** Standard deviation.
  - **sdNr:** Standard deviation, normalized.
  - **other:** Other aggregation method. Add description to aggregationOther.

- **aggregationOther:** Description for other type of aggregation not listed in aggregation.
- **aggregationDesc:** Information on or reference to the measurements that were included to calculate the aggregated measurement that is being reported.
- **value:** The actual value that is being reported for this measurement.
- **unit: The engineering unit of the measurement**.
  - **MG/D:** Million imperial gallons per day.
  - **ML/D:** Million liters per day.
  - **L/s:** Liters per second.
  - **mg/L:** Milligrams per liter.
  - **m3/h:** Cubic meters per hour.
  - **m3/d:** Cubic meters per day flowrate.
  - **empty cell:** For unitless measurements like pH.
- **notes:** Any additional notes.

**WWMeasure.CSV**

- **sampleID:** (Foreign key) Links with the identified Sample.
- **labID:** (Foreign key) Links with the identified Lab that performed the analysis.
- **assayMethodID:** (Foreign key) Links with the assay method used to perform the analysis. Use instrumentID for measures that are not viral measures.
- **instrumentID:** (Foreign key) Links with the Instrument used to perform the analysis. Use assayMethodID for viral measures.
- **reporterID:** (Foreign key) Links with the reporter that is responsible for the data.
- **analysisDate:** Date the measurement was performed in the lab.
- **reportDate:** Date the data was reported. One sampleID may have updated reports based on updates to assay method or reporting standard. In this situation, use the original sampleID but updated MeasureID, reportDate and assayID (if needed).
- **fractionAnalyzed: Fraction of the sample that is analyzed**.
  - **liquid:** Liquid fraction.
  - **solid:** Solid fraction.
  - **mixed:** Mixed/homogenized sample.
- **type: The variable that is being measured on the sample, e.g., a SARS-CoV-2 gene target region (cov), a biomarker for normalisation (n) or a water quality parameter (wq)**.
  - **covN1:** SARS-CoV-2 nucleocapsid gene N1.
  - **covN2:** SARS-CoV-2 nucleocapsid gene N2.
  - **covN200:** SARS-CoV-2 nucleocapsid gene in the region of amino acids 199–202.
  - **covN3:** SARS-like coronaviruses nucleocapsid gene N3.
  - **covE:** SARS-CoV-2 gene region E.
  - **covRdRp:** SARS-CoV-2 gene region RdRp.
  - **fluA:** Influenza virus subtype A.
  - **fluB:** Influenza virus subtype B.
  - **nPMMoV:** Pepper mild mottle virus.
  - **ncrA:** Cross-assembly phage.
  - **nbrsv:** Bovine respiratory syncytial virus.
  - **rsv:** Respiratory syncytial virus (This measure should only be used if there is no measures for rsvA or rsvB).
  - **rsvA:** Respiratory syncytial virus subtype A.
  - **rsvB:** Respiratory syncytial virus subtype B.
  - **wqTS:** Total solids concentration.
  - **wqTSS:** Total suspended solids concentration.
  - **wqVSS:** Volatile suspended solids concentration.
  - **wqCOD:** Chemical oxygen demand.
  - **wqOPhos:** Ortho-phosphate concentration.
  - **wqNH4N:** Ammonium nitrogen concentration, as N.
  - **wqTN:** Total nitrogen concentration, as N.
  - **wqPh:** pH.
  - **wqCond:** Conductivity.
  - **other:** Other measurement category. Add description to categoryOther.

- **typeOther:** Description for another variable not listed in category.
- **unit: Unit of the measurement**.
  - **gcMl:** Gene copies per milliliter.
  - **gcGs:** Gene copies per gram solids.
  - **gcL:** Gene copies per liter.
  - **Ct:** Cycle threshold.
  - **mgL:** Milligrams per liter.
  - **ph:** pH units.
  - **uScm:** Micro-siemens per centimeter.
  - **tnms:** Total number of Moore swab samples.
  - **ppms:** Percent positive, for Moore swab.
  - **pps:** Percent primary sludge, for total solids.
  - **other:** Other measurement of viral copies or wastewater treatment plant parameter. Add description to UnitOther.

- **unitOther:** Description for other measurement unit not listed in unit.
- **index:** Index number in case the measurement was taken multiple times. Index is intended to link replicate values that come from the same sample being analyzed.
- **value:** The actual measurement value that was obtained through analysis.
- **CF (sample specific):** Concentration Factor (CF) is the degree to which the concentration observed in the final qPCR assay volume has been magnified compared to concentration of the analyte in the original sample (see Box 2.1 Protocol).
- **ESV (sample specific):** Effective Sample Size/Volume (ESV) is the amount of the original sample size/volume that was actually analyzed in a qPCR reaction (see Box 2.1 Protocol)
- **qualityFlag:** Input ‘ND’ to indicate non-detect (zero value), ‘J’ for value close to the lowest point on the standard curve, or ‘UJ’ for trace values. Please refer to the Protocol for additional details.
- **notes:** Any additional notes.

## Technical Validation of Data Set

RT-qPCR controls were included with each PCR run to identify potential issues with nucleic acid extraction, cDNA generation, PCR amplification, and sample processing. As described above in Table S3, several testing institutions performed “spike-in” verifications using alternative viral internal positive standards, such as 229E, to ensure that the sample nucleic acid enrichment and extraction process occurred successfully and to mitigate the risk of false negatives. Most testing institutions ran every sample collected from January 1^st^, 2021, and March 3rd, 2023, in technical triplicates, and a few ran technical duplicates instead for both SARS-CoV-2 genomic targets and PMMoV genomic targets. Inhibition in samples was mainly verified via either the “spike-in” of a known quantity of an internal viral standard material or via serial dilution and measurement of PMMoV. Several testing institutions also monitored viral signal fluctuations and drift between SARS-CoV-2 N1 and N2 genomic regions^45^. Almost all testing institutions included standard curves with every run and ensured that their R^2^ was above 0.95 and that the PCR reaction efficiency was between 90–110%. Many testing institutions also included periodical RNA concentration checks in samples using spectrophotometric (NanoDrop - ThermoFisher, Waltham, MA) or fluorometric (Qubit – ThermoFisher, Waltham, MA) methodologies to ensure no significant changes in nucleic acid extraction efficiency occurred over time.

### Limitations inherent to the dataset

The Ontario WSI SARS-CoV-2 dataset is subject to notable variability that may occur during the sampling of the wastewaters, the analyses of the samples, and/or the subsequent data analyses^44^. This variability could stem from factors such as operational fluctuations within sewersheds (such as the use of sewer gates or underground storage containers to minimize sewer overflows or the use of sewers for wastewater storage during WRRF maintenance), at WRRFs (such as equipment shutdowns that affect the operation of screens, grit-removal units or primary sludge clarifiers) which might impact the sample collection efficiency. Moreover, the dataset encompasses wastewater samples from diverse sites and WRRFs throughout Ontario^44^. Each of these sites has a unique source of water, industrial influence, and inflow and infiltration sources, which may significantly impact the wastewater’s physical, chemical, or biological characteristics. Such variations can introduce inhibitors that impact the efficiency of nucleic acid extraction and RT-qPCR analysis, key steps in detecting and quantifying SARS-CoV-2 in wastewater. These factors likely contribute to differences in the dataset, complicating the interpretation of trends and patterns related to the incidence and dynamics of SARS-CoV-2 across Ontario. Drawing direct and uniform conclusions about the virus’ spread and intensity in different regions is complicated by these potential disparities. Efforts to standardize sampling protocols and address potential sources of bias could enhance the robustness of future analyses and interpretations of the dataset, providing more accurate insights into the behavior of SARS-CoV2 within the province^44^.

## Usage Notes

When integrated with other types of epidemiological data, WBS can contribute to a more holistic understanding of disease incidence at both the provincial and national levels in Canada. The extensive dataset and comprehensive methodology outlined in this manuscript, which includes specific normalization techniques, is not only instrumental in improving the current understanding of SARS-CoV-2 WBS but also holds promise for helping public health units and researchers make better predictions for future outbreaks of similar viral diseases. This set of protocols can be adapted by other research institutions or public health agencies interested in employing WBS.

Given the high costs and logistical challenges associated with gathering longitudinal and geospatial data, effective strategies for data preservation are of paramount importance. Transparency in sharing data related to the COVID-19 pandemic remains a critical factor for enabling informed policy decisions and ensuring public trust. Open access to datasets can facilitate retrospective studies that correlate WBS-acquired viral incidence in communities with the timeline of specific policy implementations and their outcomes. Given that COVID-19 has now been a major public health event for several years and has manifested a range of long-term health effects, the data collected through initiatives like Ontario’s WSI becomes even more invaluable. This information can assist healthcare professionals and researchers in investigating correlations between areas with high viral transmission and the occurrence of specific long-term medical conditions, thereby deepening our understanding of the pandemic’s ongoing health impacts.

### Data usage by public health units

WBS data offers valuable insights into the incidence of SARS-CoV-2 within a region. In Ontario, the WSI dataset played a critical role, evident through its adoption by all 34 public health units in the province^44^. Typical usages of the WSI dataset involved the generation of graphs for public dissemination and communications on public health unit websites and the internal review of the dataset by public health units to inform public health communications and provide weekly updates to the Province of Ontario’s Medical Officer of Health^44^.

The significance of WBS data across the province increased by most public health units after December 2021, when the Province of Ontario modified its clinical PCR screening criteria for its residents, similar to many other jurisdictions. As a result of these criteria changes, clinical SARS-CoV-2 PCR testing capacity saw a considerable decrease, primarily restricted to inpatients across all 34 public health units. As a result, WBS became a valuable complement to help track disease incidence and viral transmission dynamics across the province. The shift from clinical indicators to WBS was crucial in assessing the overall respiratory disease burden in various communities, with a notable shift in WBS data being used as the leading indicator of respiratory disease in Ottawa. Where Ottawa Public Health is currently weighing WBS data at 40% of their calculated overall assessment of respiratory disease in the nation’s capital city^45^.

Public Health Ontario also employed the WBS data to publicly disseminate aggregated, regional SARS-CoV-2 signals to the public through their website from^46^ December 1^st^, 2021 onward. This further reinforces the increasing reliance on WBS data for monitoring and responding to the COVID-19 pandemic across Ontario.

### Supplementary information

Supplemental Tables 1-3

## Data Availability

The analytical codes developed during this project are openly accessible on Zenodo^44^ and in our GitHub repository (https://github.com/OntarioWastewaterSurveillanceConsortium/sars-cov-2-data) and are shared under the terms of the Creative Commons (CC) BY License.
