# Supplementary material for: SARS-CoV-2 viral titer measurements in Ontario, Canada wastewaters throughout the COVID-19 pandemic
Source: Sci Data. 2024 Jun 21;11:656. doi: 10.1038/s41597-024-03414-w (PMC11192951; doi:10.1038/s41597-024-03414-w)
Supplement: Supplementary file 1 — Supplemental Tables 1-3 [file 41597_2024_3414_MOESM1_ESM.docx]

SARS-CoV-2 viral titer measurements in Ontario, Canada wastewaters throughout the COVID-19 pandemic

Patrick M. D’Aoust^1^*, Nada Hegazy^1^*, Nathan T. Ramsay^1^, Minqing Ivy Yang^2^, Hadi A. Dhiyebi^3^, Elizabeth Edwards^2^, Mark R. Servos^3^, Gustavo Ybazeta^8^, Marc Habash^4^, Lawrence Goodridge^4^, Art Poon^5^, Eric Arts^5^, R. Stephen Brown^6^, Sarah Jane Payne^6^, Andrea Kirkwood^7^, Denina Simmons^7^, Jean-Paul Desaulniers^7^, Banu Ormeci^9^, Christopher Kyle^10^, David Bulir^11^, Trevor Charles^3^, R. Michael McKay^12^, Kimberley Gilbride^13^, Claire Oswald^13^, Hui Peng^2^, Vince Pileggi^14^, Menglu L. Wang^13^, Arthur Tong^13^, Diego Orellano^13^, *WSI Consortium*, Christopher T. DeGroot^5^, Robert Delatolla^1^

1: University of Ottawa, Ottawa, ON, Canada

2: University of Toronto, Toronto, ON, Canada

3: University of Waterloo, Waterloo, ON, Canada

4: University of Guelph, Guelph, ON, Canada

5: Western University, London, ON, Canada

6: Queen’s University, Kingston, ON, Canada

7: Ontario Tech University, Oshawa, ON, Canada

8: Health Sciences North Research Institute, Sudbury, ON, Canada

9: Carleton University, ON, Ottawa, Canada

10: Trent University, ON, Peterborough, Canada

11: McMaster University, Hamilton, ON, Canada

12: University of Windsor, Windsor, ON, Canada

13: Toronto Metropolitan University, Toronto, ON, Canada

14: Ontario Ministry of the Environment, Conservation and Parks

* Contributed equally

Corresponding author: Dr. Robert Delatolla

**Table S1: List of testing institutions, sampled WRRFs, and sampling site characteristics**

This table describes in detail the WRRF location and characteristics, and the type of sampling performed as part of the Ontario WSI, organized by testing institution. Testing institution names are listed according to their respective sampling sites located in Northern, Easter, Central, and Western, Ontario and are color-coordinated and match colors in the visualization in Figure 1.

| Testing institution & Region | WRRF | Wastewater sample type | Sampling method (*A, **P or ***G) | Sample  processing  volume (mL) | Fixed sample processing mass of solids (mg) - if applied | Sampling start and end date | Sewered population per WRRF/upstream sampling site | Average yearly flow (min-max) of WRRF/upstream sampling site (MLD) | Coordinates (Lat., Long.) of WRRF/upstream sampling site | |
| --- | --- | --- | --- | --- | --- | --- | --- | --- | --- | --- |
| HSNRI - Northern | Haileybury Wastewater Treatment Facility | Influent | A | 40 | N/A | November 11^th^, 2022 - present | 2,303 | 1.2 | 47.4449 | -79.6301 |
|  | Kapuskasing Wastewater Treatment Plant | Influent | A | 40 | N/A | June 22^nd^, 2021 - present | 8,057 | 5.8 | 49.4241 | -82.4383 |
|  | Kirkland Lake Wastewater Treatment Plant | Influent | A | 40 | N/A | October 21^st^, 2022 – present | 75,55 | 9.0 | 48.1359 | -80.0411 |
|  | Mattagami Wastewater Treatment Plant | Influent | A | 40 | N/A | June 23^rd^, 2021 - present | 30,580 | 21.0 | 48.493 | -81.358 |
|  | Moosonee Wastewater Treatment Plant (Main Pumping Station) | Influent | A | 40 | N/A | July 19^th^, 2021 - Jan 1^st^, 2023 | 683 | 1.1 | 51.247 | -80.6761 |
|  | North Bay Wastewater Treatment Plant | Influent | A | 40 | N/A | June 1^st^, 2021 - present | 50,352 | 36.0 | 46.3031 | -79.4628 |
|  | Sault Ste. Marie East End Wastewater Treatment Plant | Influent | A | 40 | N/A | May 31^st^, 2021 - present | 52,087 | 30.4 | 46.506 | -84.2574 |
|  | Greater Sudbury Wastewater Treatment Plant | Influent | A | 40 | N/A | January 25^th^, 2021 - present | 94,296 | 50.0 | 46.4656 | -81.0356 |
| Carleton University - Eastern | Brockville Wastewater Treatment Plant | Influent | A | 30 | N/A | May 26^th^, 2021 - present | 22,546 | 16.4 (10.9 - 43.8) | 44.6034 | -75.6635 |
|  | Kemptville Water Pollution Control Plant | Influent | A | 30 | N/A | May 26^th^, 2021 - March 31^st^, 2023 | 4,039 | 4.5 | 45.0286 | -75.6446 |
|  | Pembroke Pollution Control Centre | Influent | A | 30 | N/A | June 6^th^, 2021 - present | 16,152 | 16.0 | 45.8250 | -77.0834 |
|  | Petawawa Wastewater Treatment Facility | Influent | A | 30 | N/A | June 4^th^, 2021 - present | 12,560 | 8.7 | 45.9005 | -77.2915 |
|  | Smiths Falls Wastewater Treatment Plant | Influent | A | 30 | N/A | January 26^th^, 2022 - present | 12,560 | 4.7 | 44.8932 | -76.0009 |
| Queen’s University – Eastern | Ravensview Wastewater Treatment Plant (Kingston East and Central) | Influent | A | 240 | <250 | February 18^th^, 2021 - present | 63,928 | 57.3 | 44.2413 | -76.4206 |
|  | King Street Pumping Station - Kingston Sewershed | Influent | A | 240 | <250 | March 25^th^, 2021 - April 6, 2023 | 13,351 | N/A | 44.2227 | -76.4934 |
|  | Cataraqui Bay Wastewater Treatment Plant | Post-grit influent | A | 120 | <250 | February 18^th^, 2021 - present | 52,084 | 29.3 | 44.2140 | -76.5507 |
|  | Morrisburg Wastewater Treatment Plant | Post-grit influent | A | 120 | <250 | June 15th, 2022 - March 30^th^, 2023 | 2,294 | 2.02 | 44.8999 | -75.1684 |
|  | Casselman Township Pumping Station | Influent | A | 120 | <250 | May 16^th^, 2021 - March 30^th^, 2023 | 2,383 | 1.13 | 45.3168 | -75.0913 |
|  | Cornwall Wastewater Treatment Plant | Post-grit influent | A | 120 | <250 | March 11^th^, 2021 - present | 47,459 | 44.0 | 45.0293 | -74.6779 |
|  | Hawkesbury Water Pollution Control Plant | Influent | A | 120 | <250 | July 16^th^, 2021 -  present | 10,302 | 13.8 | 45.6117 | -74.5961 |
|  | Loyalist Township (Amherstview) Water Pollution Control Plant | Post-grit influent | A | 120 | <250 | May 5^th^, 2021 - April 4^th^, 2023 | 9,832 | 3.39 | 44.2331 | -76.6610 |
|  | Wallaceburg Wastewater Treatment Plant | Post-grit influent | A | 120 | <250 | January 22^nd^, 2022 - present | 10,959 | 8.4 | 42.5808 | -82.3884 |
| Trent University - Eastern | Belleville Water Pollution Control Plant | Influent | A | 80 | N/A | February 2^nd^, 2021 -present | 46,926 | 27.9 (11.4 - 115.9) | 44.1583 | -77.3729 |
|  | Havelock Wastewater Treatment Plant | Influent | A | 80 | N/A | December 2^nd^, 2021 - March 27^th^, 2023 | 937 | 0.8 (0.5 - 1.5) | 44.3763 | -77.9801 |
|  | Lakefield Wastewater Treatment Plant | Post-grit influent | A | 80 | N/A | October 15^th^, 2021 - March 31^st^, 2023 | 1,940 | 1.4 | 44.4178 | -78.2598 |
|  | Millbrook Wastewater Treatment Plant | Post-grit influent | A | 80 | N/A | June 7^th^, 2021 -present | 2,103 | 1.0 (0.6 - 2.2) | 44.1565 | -78.4442 |
|  | Norwood Wastewater Treatment Plant | Influent | A | 80 | N/A | November 30^th^, 2021 - March 23^rd^, 2023 | 1,913 | 0.5 | 44.4225 | -77.8779 |
|  | Peterborough Wastewater Treatment Plant | Influent | A | 80 | N/A | January 18^th^, 2021 -present | 87,971 | 39.3 (31.2 - 76.9) | 44.2767 | -78.3181 |
| University of Ottawa - Eastern | Ottawa Robert O. Pickard Environmental Centre Water Pollution Control Plant | Primary sludge | G | 40 | 250 | July 31^st^, 2020 - present | 960,254 | 405.9 (249.0 - 1,068.0) | 45.4619 | -75.5903 |
|  | Ottawa Sewershed #1  (Ledbury-Heron Gate-Ridgemont-Sheffield Glen neighborhood) | Influent | A | 40 | 250 | March 7^th^, 2021 - November 15^th^, 2022 | 15,335 | 1.4 (0.6 - 2.1) | 45.39504 | -75.6026 |
|  | Ottawa Sewershed #2 (Jasmine Park-Sawmill Creek-Beacon Hill neighborhood) | Influent | A | 40 | 250 | March 12^th^, 2021 - November 15^th^, 2022 | 7,993 | 2.4 (1.6 - 4.8) | 45.44202 | -75.5917 |
|  | Ottawa Sewershed #3  (Overbrook-McArthur-Hurdman neighbourhood) | Influent | A | 40 | 250 | March 7^th^, 2021 - November 15^th^, 2022 | 21,213 | 3.4 (1.4 - 3.2) | 45.42534 | -75.6335 |
|  | ^1^Hamilton Woodward Wastewater Treatment Plant | Primary sludge | G | 40 | 250 | July 14^th^, 2020 - present | 512,000 | 263.2 (190.5 - 559.1) | 43.2525 | -79.7715 |
|  | ^1^Hamilton Dundas Wastewater Treatment Plant | Primary sludge | G | 40 | 250 | July 7^th^, 2021 - November 23^rd^, 2022 | 47,868 | 18.2 | 43.2675 | -79.9438 |
| Ontario Tech University - Central | Ajax Bayly Pumping Station | Influent | A | 30 | N/A | January 6^th^, 2021 - March 30^th^, 2023 | 146,563 | 29.4 (26.1 -76.4) | 43.8383 | -79.0418 |
|  | Barrie Wastewater Treatment Plant | Influent | A | 30 | N/A | March 18^th^, 2021 - present | 158,992 | 57.7 (52.0-79.0) | 44.3760 | -79.6901 |
|  | Bracebridge - Lagoon Lane Wastewater Treatment Plant | Influent | A | 30 | N/A | May 9^th^, 2022 - March 29^th^, 2023 | 10,240 | 3.9 | 45.0200 | -79.3180 |
|  | Collingwood Water Pollution Control Plant | Influent | A | 30 | N/A | March 23^rd^, 2021 - March 29^th^, 2023 | 21,793 | 18.1 | 44.5040 | -80.2234 |
|  | Corbett Creek Water Pollution Control Plant | Influent | A | 30 | N/A | March 18^th^, 2021- present | 156,719 | 55.5 (42.0-100.7) | 43.8554 | -78.8900 |
|  | Courtice Water Pollution Control Plant | Influent | A | 30 | N/A | March 18^th^, 2021 - March 30^th^, 2023 | 146,472 | 40.7 (25.0 - 99.3) | 43.8713 | -78.7560 |
|  | Pickering Durham Liverpool Pumping Station | Influent | A | 30 | N/A | January 6^th^, 2021-March 30^th^, 2023 | 69,191 | 21.3 (16.0 - 53.0) | 43.8147 | -79.0793 |
|  | Harmony Creek Water Pollution Control Plant | Influent | A | 30 | N/A | March 18^th^, 2021 - March 30^th^, 2023 | 41,554 | 18.5 (12.8 - 28.1) | 43.8794 | -78.8234 |
|  | Midland Wastewater Treatment Plant | Influent | A | 30 | N/A | March 19^th^, 2021 - present | 16,979 | 7.7 | 44.7570 | -79.8740 |
|  | Newcastle Water Pollution Control Plant | Influent | A | 30 | N/A | March 18^th^, 2021 - March 30^th^, 2023 | 11,648 | 3.3 (2.3 - 8.5) | 43.8990 | -78.5940 |
|  | City of Orillia Wastewater Treatment Centre | Influent | A | 30 | N/A | March 19^th^, 2021 - March 30^th^, 2023 | 29,613 | 15.4 (9.2 - 24.4) | 44.5900 | -79.4110 |
|  | Town of Penetanguishene Wastewater Pollution Control Plant | Influent | A | 30 | N/A | May 19^th^, 2022 - March 26^th^, 2023 | 8,960 | 3.0 | 44.7720 | -79.9390 |
|  | Port Darlington Water Pollution Control Plant | Influent | A | 30 | N/A | March 18^th^, 2021 - present | 45,454 | 13.8 (10.1 - 29.4) | 43.8932 | -78.6632 |
| Toronto Metropolitan University – Central | Humber Water Pollution Control Plant | Post-screen influent | A | 40 | 100-150 | November 23^rd^, 2020 - present | 701,639 | 255.6 (189.4 - 529.3) | 43.6336 | -79.4768 |
|  | Toronto Sewershed (site 1a) | Influent | A | 200 | 100-150 | April 6^th^, 2021 - March 30^th^, 2023 | 135,923 | 54.8 (41.8 - 118.8) | 43.7137 | -79.5400 |
|  | Toronto Sewershed (site 1b) | Influent | A | 200 | 100-150 | January 20^th^, 2021 - March 30^th^, 2023 | 52,481 | 33.4 (30.8 - 57.8) | 43.7301 | -79.5496 |
|  | Toronto Sewershed (site 2a) | Influent | A | 200 | 100-150 | December 7^th^, 2020 - March 30^th^, 2023 | 64,705 | 21.7 (4.9 - 34.9) | 43.7358 | -79.4958 |
|  | Toronto Sewershed (site 2b) | Influent | A | 200 | 100-150 | January 4^th^, 2021 - March 30^th^, 2023 | 5,443 | 2.0 (1.1 - 11.2) | 43.7619 | -79.5084 |
|  | Toronto Sewershed (site 4) | Influent | A | 200 | 100-150 | December 7^th^, 2020 - March 30^th^, 2023 | 3,971 | 3.8 (2.1 - 5.1) | 43.6567 | -79.5388 |
|  | Toronto Sewershed (site 5) | Influent | A | 200 | 100-150 | January 13^thth^, 2021 - March 30^th^, 2023 | 2,733 | N/A | 43.6356 | -79.5067 |
| University of Toronto - Central | Acton Wastewater Treatment Plant | Post-grit influent | A | 120 (September 8^th^, 2021 - October 12^th^, 2021)  40 (after October 12^th^, 2021) | <250 | September 8^th^, 2021 - March 31^st^, 2023 | 10,477 | 5.2 | 43.6287 | -80.0222 |
|  | Ashbridge Wastewater Treatment Plant | Post-screen influent | A | 80 | <250 | February 11^th^, 2021 - present | 1,506,692 | 570.4 | 43.6569 | -79.3194 |
|  | Oakville Southeast Wastewater Treatment Plant | Post-grit influent | A | 40, 60, or 120 (depending on the inhibition) | <250 | September 8^th^, 2021 - March 31^st^, 2023 | 41,972 | 21.9 | 43.4133 | -79.6939 |
|  | Georgetown Wastewater Treatment Plant | Post-grit influent | A | 120 (September 8^th^, 2021 - October 18^th^, 2021)  60 (after October 20^th^, 2021) | <250 | September 8^th^, 2021 - March 31^st^, 2023 | 42,101 | 12.5 | 43.6391 | -79.8784 |
|  | Highland Creek Wastewater Treatment Plant | Post-screen influent | A | 80 | <250 | February 11^th^, 2021 - present | 536,103 | 163.3 | 43.7665 | -79.1490 |
|  | Mid-Halton Wastewater Treatment Plant | Post-grit influent | A | 80 prior to September 1^st^, 2021; afterwards 120 | <250 | September 8^th^, 2021 - March 31^st^, 2023 | 220,454 | 169.0 | 43.4205 | -79.7315 |
|  | Skyway Wastewater Treatment Plant | Post-grit influent | A | 80 prior to September 1^st^, 2021; afterwards 120 | <250 | September 8^th^, 2021 - present | 190,359 | 7.7 | 44.7571 | -79.8762 |
|  | North Toronto Wastewater Treatment Plant | Post-screen influent | A | 120 | <250 | February 11^th^, 2021 - March 31^st^, 2023 | 193,958 | 17.5 | 43.6991 | -79.3564 |
|  | Oakville Southwest Wastewater Treatment Plant | Post-grit influent | A | 200 | <250 | September 8^th^, 2021 - March 31^st^, 2023 | 49,855 | 27.5 | 43.4133 | -79.6939 |
| University of Waterloo - Central | Clarkson Water Pollution Control Plant | Post-grit influent | A | 40 | N/A<250 | January 1^st^, 2021 - March 31^st^, 2023 | 643,331 | 206.5 | 43.4960 | -79.6191 |
|  | Humber Air Management Facility Pumping Station | Post-grit influent | A | 40 | N/A <250 | January 14^th^, 2021 - March 31^st^, 2023 | 105,270 | 21.7 (6.7 - 41.3) | 43.74991 | -79.6393 |
|  | Galt Wastewater Treatment Plant | Post-grit Influent | A | 40 | N/A <250 | January 16^th^, 2021 - March 31^st^, 2023 | 89,714 | 28.1 | 43.3406 | -80.3152 |
|  | GE Booth Wastewater Treatment Plant | Post-grit influent | A | 40 | N/A <250 | January 14^th^, 2021 - March 31^st^, 2023 | 1,089,738 | 432.0 | 43.5784 | -79.5476 |
|  | Kitchener Wastewater Treatment Plant | Post-grit influent | A | 40 | N/A <250 | January 16^th^, 2021 - March 31^st^, 2023 | 256,153 | 70.0 | 43.3991 | -80.4218 |
|  | Waterloo Wastewater Treatment Plant | Influent | A | 40 | N/A <250 | January 16^th^, 2021 - March 31^st^, 2023 | 122,823 | 41.4 | 43.4878 | -80.5075 |
|  | Newmarket Pumping Station - York Region | Influent | A | 40 | N/A <250 | January 20^th^, 2021- November 10^th^, 2021 | 100,428 | 20.2 (10.8 - 36.7) | 44.0729 | -79.4533 |
|  | Leslie Street Pumping Station - York Region | Influent | G | 40 | N/A <250 | February 8^th^, 2021 - March 31^st^, 2023 | 295,232 | 59.9 (6.9 - 119.3) | 43.8127 | -79.3737 |
|  | Warden and 407 Pumping Station - York Region | Influent | G | 40 | N/A <250 | April 26^th^, 2021 - March 31^st^, 2023 | 650,303 | 51.2 (11.7 - 82.5) | 43.8457 | -79.3350 |
| University of Guelph – Western | Fort Erie Anger Avenue Sewage Treatment Plant | Influent | A | 350 | 200 | May 26^th^, 2021 - March 31^st^, 2023 | 16,638 | 14.0 (5.1 - 55.4) | 42.9381 | -78.9268 |
|  | Grimsby Baker Road Wastewater Treatment Plant | Influent | A | 350 | 200 | May 28^th^, 2021 - March 31^st^, 2023 | 53,144 | 19.9 (10.0 - 60.9) | 43.1940 | -79.5376 |
|  | Fort Erie Crystal Beach Wastewater Treatment Plant | Influent | A | 350 | 200 | May 26^th^, 2021 - March 31^st^, 2023 | 9,053 | 5.47 (1.97 - 22.9) | 42.8618 | -79.0575 |
|  | Guelph Wastewater Treatment Plant | Influent | A | 350 | 200 | November ^17th^, 2020 - March 31^st^, 2023 | 144,789 | 64.0 | 43.5250 | -80.2644 |
|  | Orangeville Wastewater Treatment Plant | Influent | A | 350 | 200 | February 22^nd^, 2021 - March 31^st^, 2023 | 32,601 | 11.7 | 43.9181 | -80.0870 |
|  | St. Catharines Port Weller Sewer Treatment Plant | Influent | A | 350 | 200 | May 26^th^, 2021 - March 31^st^, 2023 | 79,496 | 36.7 (19.2 - 138.8) | 43.2259 | -79.2183 |
|  | Queenston Wastewater Treatment Plant | Influent | A | 350 | 200 | May 26^th^, 2021 - March 31^st^, 2023 | 279 | 0.16 (0.05 - 1.24) | 43.1632 | -79.0498 |
|  | Niagara Falls Stamford Wastewater | Influent | A | 350 | 200 | May 26^th^, 2021 - March 31^st^, 2023 | 94,304 | 39.0 (18.1 - 155.8) | 43.1242 | -79.0855 |
|  | Welland Wastewater Treatment Plant | Influent | A | 350 | 200 | May 28^th^, 2021 - March 31^st^, 2023 | 67,448 | 36.9 (16.5 - 1134) | 43.0076 | -79.2378 |
| McMaster University - Western | ^2^Brantford Water Treatment Plant | Post-grit influent | A | 32 | N/A | October 5^th^, 2021 - Present | 105,122 | 36.0 (31.2 - 40.4) | 43.1286 | -80.2310 |
|  | ^2^Cobourg Water Pollution Plant | Post-grit influent | A | 32 | N/A | October 20^th^, 2021 - Present | 13,149 | 7.9 (6.7 - 9.7) | 43.9606 | -78.1824 |
|  | ^2^Kawartha Lindsay Wastewater Treatment Plant | Post-grit influent | A | 32 | N/A | August 17^th^, 2021 - Present | 23,794 | 10.9 (6.6 - 17.2) | 44.3869 | -78.7393 |
|  | ^2^Minden Wastewater Treatment Facility | Post-grit influent | A | 32 | N/A | August 17^th^, 2021 - April 28^th^, 2023 | 2,858 | 0.5 (0.5 - 0.7) | 44.9170 | -78.7325 |
|  | Craigleith Water Treatment Plant | Post-grit influent | A | 32 | N/A | October 6^th^, 2021 - Present | 2,173 | 3.3 (2.5 - 4.2) | 44.5270 | -80.2938 |
|  | Goderich Water Pollution Control Plant | Post-grit influent | A | 32 | N/A | August 18^th^, 2021 - Present | 8,192 | 6.6 (3.1 - 19.0) | 43.7308 | -81.7234 |
|  | Owen Sound Water Treatment Plant | Post-grit influent | A | 32 | N/A | October 13^th^, 2021 - Present | 23,113 | 15.0 (10.7 - 30.1) | 44.5846 | -80.9329 |
|  | St. Thomas Water Pollution Control Plant | Influent | A | 32 | N/A | October 5^th^, 2021 - Present | 46,011 | 48.7 (38.2 - 62.0) | 42.7689 | -81.2046 |
|  | Stratford Water Pollution Control Plant | Post-grit influent | A | 32 | N/A | August 19^th^, 2021 - Present | 32,469 | 20.5 (18.0 - 22.3) | 43.3693 | -81.0040 |
| Western University - Western | Greenway Wastewater Treatment Plant | Influent | A | 40 | N/A | March 22^nd^, 2021 - present | 183,346 | 120.0 (78.7 - 217.9) | 42.9746 | -81.2823 |
|  | London Adelaide Wastewater Treatment Plant | Post-grit influent | A | 40 | N/A | March 22^nd^, 2021- March 30^th^, 2023 | 74,274 | 22.0 (15.0 - 55.0) | 43.0161 | -81.2483 |
|  | Caledonia Wastewater Treatment Plant | Influent | A | 40 | N/A | June 15^th^, 2021 - present | 11,291 | 2.8 | 43.0684 | -79.9457 |
|  | Hagersville Wastewater Treatment Plant | Post-grit influent | A | 40 | N/A | June 15^th^, 2021 - November 22^nd^, 2022 | 4,107 | 2.7 | 42.9546 | -80.0610 |
|  | ^3†^London Oxford Pollution Control Plant | Post-grit influent | A | 40 | N/A | March 22^nd^, 2021 - March 30^th^, 2023 | 44,693 | 11.1 (8.6 - 22.3) | 42.9686 | -81.3426 |
|  | ^†^Pottersburg Wastewater Treatment Plant | Post-grit influent | A | 40 | N/A | March 22^nd^, 2021 - March 30^th^, 2023 | 41,751 | 22.6 (14.2 - 41.8) | 42.9734 | -81.1792 |
|  | Sarnia Water Pollution Control Centre | Post-grit influent | A | 40 | N/A | June 10^th^, 2021 - present | 65,038 | 26.9 (20.1 - 75.0) | 42.9551 | -82.4052 |
|  | London Vauxhaul Pollution Control Plant | Influent | A | 40 | N/A | March 22^nd^, 2021 - present | 26,659 | 13.1 (6.7 - 49.2) | 42.9736 | -81.2051 |
|  | Woodstock Wastewater Treatment Plant | Influent | A | 40 | N/A | May 9^th^, 2021 - present | 50,271 | 20.4 (11.5 - 67.8) | 43.1366 | -80.7716 |
|  | Simcoe Water Pollution Control | Post-grit influent | A | 40 | N/A | October 25^th^, 2022 - present | 416,734 | 8.0 (5.6 - 18.7) | 42.8294 | -80.2960 |
| University of Windsor - Western | ^3^Sault Ste. Marie East End Wastewater Treatment Plant | Influent | A | 60 - 80 | N/A | February 8^th^, 2021 - May 25^th^, 2021 | 52,087 | 30.4 | 46.5057 | -84.2574 |
|  | ^3^Thunder Bay Atlantic Ave Water Pollution Control Plant | Influent | A | 60 - 80 | N/A | February 1^st^, 2021 - present | 97,524 | 65.0 (28.6 - 320.8) | 48.3970 | -89.2260 |
|  | Amherstburg Wastewater Treatment Plant | Influent | A | 60 - 80 | N/A | December 14^th^, 2020 - March 27^th^, 2023 | 18,276 | 13.1 (2.6 - 23.7) | 42.0938 | -83.1091 |
|  | Chatham Water Pollution Control Plant | Influent | A | 60 - 80 | N/A | May 10^th^, 2021 - present | 46,674 | 22.5 (11.7 - 63.7) | 42.3894 | -82.2144 |
|  | Denis St. Pierre Wastewater Treatment Plant | Influent | A | 60 - 80 | N/A | December ^23rd^, 2020 - March 30^th^, 2023 | 25,847 | 20.3 (7.3 - 32.24) | 42.2907 | -82.7343 |
|  | Leamington Pollution Control Center | Influent | A | 60 - 80 | N/A | December 9^th^, 2020 - present | 21,255 | 13.0 (7.1 - 41.3) | 42.0345 | -82.5872 |
|  | Little River Pollution Control Plant | Influent | A | 60 - 80 | N/A | December 4^th^, 2020 - March 31^st^, 2023 | 89,329 | 96.0 (2.0 - 190.0) | 42.3292 | -82.9268 |
|  | Lou Romano Water Reclamation Plant | Influent | A | 60 - 80 | N/A | October 19^th^, 2020 - present | 179,209 | 65.0 (38.5 - 500.0) | 42.2833 | -83.0864 |
|  | North Bay Wastewater Treatment Plant | Influent | A | 60 - 80 | N/A | February 9^th^, 2021 - May 26, 2021 | 50,352 | 36.0 | 46.3031 | -79.4628 |
|  | Greenway Wastewater Treatment Centre | Influent | A | 60 - 80 | N/A | December 14^th^, 2020 - March 21^st^, 2021 | 183,346 | 120.0 (78.7 - 217.9) | 42.9750 | -81.2823 |
|  | Adelaide Wastewater Treatment Plant | Influent | A | 60 - 80 | N/A | December 14^th^, 2020 - March 21^st^, 2021 | 74,274 | N/A | 43.0162 | -81.2483 |
|  | ^3†^London Oxford Pollution Control Plant | Influent | A | 60 - 80 | N/A | December14^th^, 2020 - March 21^st^, 2021 | 44,693 | 11.1 (8.6 - 22.3) | 42.9686 | -81.3426 |
|  | Vauxhall Pollution Control Plant | Influent | A | 60 - 80 | N/A | December 13^th^, 2020 - March 21^st^, 2021 | 26,659 | N/A | 42.9736 | -81.2051 |
|  | ^†^Pottersburg Wastewater Treatment Plant | Influent | A | 60 - 80 | N/A | December 14^th^ July 16^th^, 2020 - March 21^st^, 2021 | 41,751 | 22.6 (14.2 - 41.8) | 42.9734 | -81.1792 |

1: Sites located in Western Ontario region

2: Sites located in Central Ontario region

3: Sites located in Northern Ontario region

*A = Auto sampling

**P = Passive sampling

***G = Grab sampling

****NA = Not applied (used to describe processes where a fixed mass of solids was not applied)

^†^: Sites were tested by one or more testing institutions

**Table S2:** **Analytical methods: RNA enrichment, RNA extraction and RT-qPCR protocol**

This table describes the analytical methods employed by each of the testing institutions. Specifically, RNA enrichment method, RNA extraction/isolation methods, and RT-qPCR conditions with specific gene targets are described for both SARS-CoV-2 targets and PMMoV. Testing institution names are listed according to their respective sampling sites located in Norther, Easter, Central, and Western, Ontario and are color-coordinated and match colors in the visualization in Figure 1.

|  | RNA enrichment | | RNA extraction | | RT-qPCR | | | | | | | | | | |
| --- | --- | --- | --- | --- | --- | --- | --- | --- | --- | --- | --- | --- | --- | --- | --- |
|  |  |  |  |  |  | |  | SARS-CoV-2 | | | | Normalization target | | | |
| Testing institution & Region | Sample pre-processing | Filtration/centrifugation processing | Extraction kit | Modification to extraction kit | PCR instrument | Supermix | Standardized materials used in assays | Targeted gene regions | RNA template volume (µL) loaded into PCR wells | Total Rt-qPCR reaction volume (µL) loaded into PCR wells | RT-qPCR cycling conditions | Normalization target | RNA template volume (µL) loaded into PCR wells | Total RT-qPCR reaction volume (µL) loaded into PCR wells | RT-qPCR cycling conditions |
| HSNRI - Northern | March 24, 2021-November 16, 2021: Reacted (PEG, NaCl, Homogenized and Mixed, 4°C)  November 17,2021-present: Homogenized and Mixed | Centrifuged (13,000 x g, 1.5 hrs min, 4°C);  March 24, 2021-November 16, 2021: PEG (Polyethylene Glycol)  November 17,2021-May 23, 2023:  PowerMicrobiome® Without PEG  May 24, 2023-present  PowerFecal Pro® | RNeasy PowerMicrobiome (Qiagen)  *Post discontinuation:*  PowerFecal Pro (Qiagen) | Lysis Step (100 µL Chloroform/Phenol/Isoamyl, 10 µL BME, DNase Skipped) | QIAquant (Qiagen)  AriaMX (Agilent) | Fast Virus One-Step Master Mix (Thermo Fisher) | Exact Diagnostics (EDX) SARS-CoV-2 Standard COV019 (Bio-Rad) | CDC N1  CDC N2 | N1: 5  N2: 5 | N1: 20  N2: 20 | 25°C 2 min  50°C 15 min  95°C 2 min  *45 cycles of*  95°C 3 sec  55°C (N1), 60°C (N2) 30 sec | PMMoV | PMMoV: 5 | PMMoV: 20 | 25°C 2 min  50°C 15 min  95°C 2 min 95°C 2 min  *45 cycles of*  95°C 3 sec  55°C 30 sec. |
| Carleton University - Eastern | Mixed by shaking 100X | Centrifuged (4200 x g, 20 min followed by 2 min, 20000 x g) | RNeasy PowerMicrobiome (Qiagen)  *Post discontinuation:*  AllPrep PowerViral DNA/RNA (Qiagen) | Protein Precipitation (20% BME at 55°C, PM1, IRS 250 µL, 10 min Incubation) | CFX96 and CFX OPUS (Bio-Rad) | Reliance One-Step (Bio-Rad) | Exact Diagnostics (EDX) SARS-CoV-2 Standard COV019 (Bio-Rad) | CDC N1  CDC N2 | N1: 5  N2: 5 | N1: 20  N2: 20 | 50°C 10 min  95°C 10 min  *45 cycles of*  95°C 10 s  60°C 30 s | PMMoV | PMMoV: 5 | PMMoV: 20 | 50°C 10 min  95°C 10 min  *45 cycles of*  95°C 10 s  60°C 30 s |
| Queens University – Eastern | Homogenized and Mixed | Centrifuged (12,000 x g, 60 min) | RNeasy PowerMicrobiome (Qiagen) | Addition of Reagents (100 µL Chloroform, 100 µL Trizol, 0.1% BME in PM1) | QIAquant® 96 5plex (Qiagen) | TaqMan Fast Virus One-Step Master Mix (Thermo Fisher) | Exact Diagnostics (EDX) SARS-CoV-2 Standard COV019 (Bio-Rad) | CDC N1  CDC N2  E | N1: 5  N2: 5  E: 5 | N1: 10  N2: 10  E: 10 | 50°C 10 min  95°C 10 min  *45 cycles of*  95°C 5 s  60°C 30 s | PMMoV | PMMoV: 5 | PMMoV: 20 | 50°C 10 min  95°C 10 min  *45 cycles of*  95°C 5 s  60°C 30 s |
| Trent University - Eastern | Mixed, Settled, and Solids Collected | Centrifuged (12,000 x g, 1 hr, 4°C); Resuspended in BME + Qiagen PM1 | AllPrep PowerViral DNA/RNA (Qiagen) on the QiaCube Connect Platform (Qiagen) | Post-Bead Processing (650-700 µL Used, No DNase, 100 µL Elution) | QuantStudio 5 (Thermo Fisher) | Reliance RT-qPCR Supermix (Bio-Rad) | Exact Diagnostics (EDX) SARS-CoV-2 Standard COV019 (Bio-Rad) | CDC N1  CDC N2 | N1: 5  N2: 5 | N1: 20  N2: 20 | 25°C 2 min  50°C 15 min  95°C 10 min  40 cycles of  95°C 10 sec  60°C 35 sec | PMMoV | PMMoV: 5 | PMMoV: 12.5 | 25°C 2 min  50°C 15 min  95°C 10 min  *40 cycles of*  95°C 10 sec  60°C 35 sec |
| University of Ottawa - Eastern | No Settling Required | Centrifuged (10,000 x g, 45 min, 4°C); decanted; Centrifuged Again (5 min) | RNeasy PowerMicrobiome (Qiagen) on QiaCube Connect Platform  *Post discontinuation:*  AllPrep PowerViral DNA/RNA (Qiagen) on QiaCube Connect Plaform | Pre-Lysis Step (Trizol, BME, PowerLyzer, 0.25 g Pellet) | CFX96 ™ (Bio-Rad) | TaqMan Fast Virus One-Step Master Mix (Thermo Fisher) | Exact Diagnostics (EDX) SARS-CoV-2 Standard COV019 (Bio-Rad) | CDC N1  CDC N2 | N1: 3  N2: 3 | N1: 10  N2: 10 | 50°C 10 min  95°C 10 min  *45 cycles of*  95°C 5 s  60°C 30 s | PMMoV | PMMoV: 1.5 | PMMoV: 10 | 50°C 10 min  95°C 10 min  *45 cycles of*  95°C 5 s  60°C 30 s |
| Ontario Tech University - Central | Homogenized, Mixed, and Reacted (PEG, Inverted, Vortexed) | Centrifuged (12,000 x g, 2 hr) | RNeasy PowerMicrobiome (Qiagen) on QiaCube Connect Platform | Pre-Lysis Step (Trizol and BME Added) | CFX96 (Bio-Rad) | Reliance RT-qPCR Supermix (Bio-Rad) | Exact Diagnostics (EDX) SARS-CoV-2 Standard COV019 (Bio-Rad) | CDC N1  CDC N2 | N1: 5  N2: 5 | N1: 15  N2: 15 | 50°C 10 min  95°C 10 mins  *45 cycles of*  95°C 10 sec  60°C 30 sec | PMMoV | PMMoV: 5 | PMMoV: 20 | 50°C 10 min  95°C 10 mins  *45 cycles of*  95°C 10 sec  60°C 30 sec |
| Toronto Metropolitan University - Central | Homogenized and Mixed | Centrifuged (12,000 x g, 50 min); Weighed Pellet; Transferred to Microcentrifuge Tube; Centrifuged Again (15 min) | AllPrep PowerViral DNA/RNA (Qiagen) using QiaCube Connect Platform | Add DNase at step 3 | CFX OPUS 384 (Bio-Rad) | Reliance RT-qPCR Supermix (Bio-Rad) | Exact Diagnostics (EDX) SARS-CoV-2 standard COV019 (BioRad) and ATCC SARS CoV2 RNA (ATCC-VR3276SD) | CDC N1  CDC N2 | N1: 5  N2: 5 | N1: 10  N2: 10 | 50°C 10 min  95°C 10 min  *45 cycles of*  95°C 30 sec  60°C 30 sec | PMMoV | PMMoV: 5 | PMMoV: 10 | 50°C 10 min  95°C 10 min  *45 cycles of*  95°C 30 sec  60°C 30 sec |
| University of Toronto - Central | Homogenize, Mixed, and Centrifuged | Centrifuged (10,000 x g, 45 min, 4°C) “low” or “=7” in the brake setting; Transferred to Microcentrifuge Tube; Centrifuged Again (13,000 x g, 1 min, 4°C) | RNeasy PowerMicrobiome (Qiagen) on QiaCube Connect Platform  *Post discontinuation:*  AllPrep PowerViral DNA/RNA (Qiagen) on QiaCube Connect Plaform | Modification (skipped DNase;, RNeasy PowerMicrobiome Consistency; 100 uL Phenol:Chloroform:Isoamyl Alcohol (25:24:1 v/v) and 10uL BME added to the lysis step) | CFX OPUS 384 (Bio-Rad) | Fast Virus One-Step (Thermo Fisher) | Concatenated Plasmid (GenBank OR994921) | CDC N1  CDC N2 | N1: 4  N2: 4 | N1: 10  N2: 10 | 50°C 5 min  95°C 2 min  *45 cycles of*  95°C 5 s  60°C 30 s | PMMoV | PMMoV: 4 | PMMoV: 10 | 50°C 5 min  95°C 2 min  *45 cycles of*  95°C 5 s  60°C 30 s |
| University of Waterloo - Central | Reacted (PEG, NaCl, Shaken, 4°C) | Centrifuged (12,000 x g, 1.5 hrs); Decanted; Centrifuged Again (5 min) | RNeasy PowerMicrobiome (Qiagen) on QiaCube Connect Platform | Pre-Lysis Step (Trizol and BME Added) | CFX96 (Bio-Rad) | TaqPath 1-Step RT-qPCR Master Mix CG (Thermo Fisher) | Exact Diagnostics (EDX) SARS-CoV-2 Standard (COV019 (BIoRaD) for SARS-CoV-2  G-block (Integrated DNA Technologie) for PMMoV | CDC N1  CDC N2 N200 | N1: 5  N2: 5  N200: 5 | N1: 20  N2: 20  N200: 20 | 25°C 2 mins  50°C 15 mins  95°C 2 mins  *45 cycles of*  95°C 3 sec  55°C (N1), 60°C (N2), 57°C (N200) 30 sec | PMMoV | PMMoV: 2.5 | PMMoV: 10 | 25°C 2 mins  50°C 15 mins  95°C 2 mins  *45 cycles of*  95°C 3 sec  55°C 30 sec |
| University of Guelph - Western | Homogenized, Mixed, and Reacted (PEG, NaCl, 4°C, 18 hrs) | Centrifuged (8,000 x g, 60 min, 4°C); Resuspended in NaPO_4_ Buffer | AllPrep PowerViral DNA/RNA (Qiagen) on the QiaCube Connect Platform | Wastewater Extraction (200 µL, 6 µL BME, 594 µL PM1) | CFX96 (Bio-Rad) | Reliance One-Step Multiplex Supermix (Bio-Rad) | Concatenated Plasmid (GenBank OR994921), provided by University of Toronto | CDC N1  CDC N2 E | N1: 5  N2: 5  E: 5 | N1: 20  N2: 20  E: 20 | 50°C 10 min  95°C 10 min  *45 cycles of*  95°C 5 s  60°C 30 s | PMMoV | PMMoV: 2.5 | PMMoV: 20 | 50°C 10 min  95°C 10 min  *45 cycles of*  95°C 5 s  60°C 30 s |
| McMaster University - Western | Filter-Concentrated (0.45 µm PES) | Mixed (AVL Buffer, Carrier RNA, BME, Trizol); Incubated (10 min); Vacuum applied; collected flow through | RNeasy PowerMicrobiome (Qiagen); PM3/PM5/PM5 Used with Vacuum Manifold  *Post discontinuation:*  AllPrep PowerViral DNA/RNA (Qiagen) | Purification Method (Vacuum Manifold, Additional PM4 Wash) | CFX96 (Bio-Rad) | Luna Probe One-Step (NEB) | Exact Diagnostics (EDX) SARS-CoV-2 Standard COV019 (Bio-Rad) | CDC N1  E | N1: 5  E: 5 | N1: 20  E: 20 | 60°C 10 min  95°C 2 min  *45 cycles of*  95°C 10 s  60°C 15 s | PMMoV | PMMoV: 5 | PMMoV: 20 | 60°C 10 min  95°C 2 min  *45 cycles of*  95°C 10 s  60°C 15 s |
| Western University - Western | Homogenized, Mixed, and Aliquoted | Before April 2023: Centrifuged (24,792 x g, 90 min)  April 2023 - May 2023: Centrifuged (4,500 x g, 20 min)  May 2023 - current: Centrifuged (4,500 x g, 120 min)  All versions: 4°C with max deceleration | RNeasy PowerMicrobiome (Qiagen)  *Post discontinuation:*  PowerFecal Pro (Qiagen) on the QiaCube Connect Platform | PowerFecal Pro: 100 µL Phenol-Chloroform-Isoamyl alcohol added to bead-beating tubes | QuantStudio 5 (Thermo Fisher) | Before May 2023: TaqPath 1-Step RT-qPCR Master Mix CG (Thermo Fisher)  Current: Luna Probe One-Step (NEB) | Concatenated plasmid provided by University of Toronto | CDC N1  CDC N2 | N1: 2.5  N2: 2.5 | N1: 10  N2: 10 | 50°C 5 min  95°C 20 sec  *45 cycles of*  95°C 15 sec  60°C 40 sec | PMMoV | PMMoV: 2.5 | PMMoV: 10 | 50°C 5 min  95°C 20 sec  *45 cycles of*  95°C 15 sec  60°C 40 sec |
| University of Windsor - Western | Filter-Concentrated (0.22 µm PES) | Filtration (Electro-negative membrane) | RNeasy PowerMicrobiome (Qiagen)  *Post discontinuation:*  AllPrep PowerViral DNA/RNA (Qiagen) | Modification (5% BME added) | MA6000 (Aumintec) | Before February 2023: Takyon Dry One-Step RT Probe MasterMix No Rox, (Eurogentec)  Current: Luna Universal Probe One-  Step (NEB) | Exact Diagnostics (EDX) SARS-CoV-2 Standard COV019 (Bio-Rad) | CDC N1  CDC N2 | N1: 5  N2: 5 | N1: 20  N2: 20 | Before Feb 7^th^. 2023:  48°C 10 min  95°C 3 min  *45 cycles of*  95°C 10 sec  60°C 55 sec  After Feb 7^th,^ 2023:  55°C 10 min  95°C 3 min  *45 cycles of*  95°C 10 sec  60°C 30 sec | PMMoV | PMMoV: 2.5 | PMMoV: 20 | Before Feb 7^th^. 2023:  48°C 10 min  95°C 3 min  *45 cycles of*  95°C 10 sec  60°C for 45 sec  After Feb 7^th,^ 2023:  55°C 10 min  95°C 3 min  *40 cycles of*  95°C 10 sec  55°C 30 sec |

**Table S3: Protocol controls, sensitivity, and quality control**

This table provides detailed information regarding the protocol controls, sensitivity checks, and quality control checks employed by each testing institution. Specifically, the assay limits of detection (ALOD) and assay limits of quantification (ALOQ), inhibition checks, any additional checks or verifications, quality assurance and quality control criteria, and additional comments, are included below, separated by testing institution. Testing institution names are listed according to their respective sampling sites located in Norther, Easter, Central, and Western, Ontario and are color-coordinated and match colors in the visualization in Figure 1.

| Testing Institution & Region | Assay limit of detection (ALOD 95%) for N1, N2, E, N200 and PMMoV (copies/reaction) | Assay limit of quantification (ALOQ 35% CV) for N1, N2, E, N200, and PMMoV (copies/reaction) | Check for inhibition | Additional check | Key QA/QC criteria | Additional comments: previous modification of the dataset |
| --- | --- | --- | --- | --- | --- | --- |
| HSNRI - Northern | N1: 4.0 copies/reaction  N2: 1.1 copies/reaction  PMMoV: 3.9 copies/reaction | N1: 9.7 copies/reaction  N2: 6.9 copies/reaction  PMMoV: 20.9 copies/ reaction | MHV spike-in concentration check. | None. | Checking the standard curve statistics,  R^2^ above 0.98, Efficiency between 90%-110%. 30 run reproducibility tracking for N1 and N2. | None. |
| Carleton University - Eastern | N1: 2.1 copies/reaction  N2: 1.8 copies/reaction;  PMMoV: 2.5 copies/reaction | N1: 10.6 copies/reaction  N2: 6.1 copies/reaction  PMMoV: 17.0 copies reaction | PMMoV dilution of 1/10. >1CT indicates inhibition | MS2 Whole process control (extraction and RT and PCR) | Repeatability and reproducibility monitoring slope, intercept, and efficiency, R^2^ above 0.975, Efficiency between 90%-110%, slope >3.1 and <3.6 | None. |
| Queens University - Eastern | N1: 4.0 copies/reaction  N2: 4.0 copies/reaction  E: 4.0 copies/reaction  PMMoV: 5.0 copies/reaction | N1: 8.0 copies/reaction  N2: 8.0 copies/reaction  E: 8.0 copies/reaction  PMMoV = 20.0 copies/well | PMMoV dilution of 1/10. | 229E spike-in concentration check. | Standard curve R^2^ and efficiency,  reliable (>95%) detection at 4 copies/well | None. |
| Trent University - Eastern | N1: 1.9 copies/reaction  N2: 1.9 copies/reaction  PMMoV: Not Measured | N1: 9.0 copies/reaction  N2: 9.0 copies/reaction PMMoV = Not Measured | PMMoV dilution of 1/10 | Batched aliquots of sludge used for long-term whole-process control | 30 run reproducibility tracking for N1, N2. N1 and N2 monitoring, outlier detection between bio-reps (re-runs the following day) | PMMoV correction factor of 10  Data post July 1^st,^ 2021 using consistent method. Different controls and instruments prior  No retroactive adjustments  Some variation between EDX Batches |
| University of Ottawa - Eastern | N1: 3.0 copies/reaction  N2: 3.0 copies/reaction  PMMoV: 3.0 copies/well | N1: 4.0 copies/well  N2: 6.0 copies/well  PMMoV = 10.0 copies/well | PMMoV dilutions of 1/10, 1/40, and no dilution for Ottawa. PMMoV dilution 1/8 and 1/16 for Hamilton. | None. | R^2^ above 0.95, Efficiency between 90%-110%, N1/N2 drifting, inhibition, contamination, snowmelt | None. |
| Ontario Tech University – Central | N1: 2.8 copies/well  N2: 5.6 copies/well  PMMoV: Not Measured | N1: 3.5 copies/reaction  N2: 10.0 copies /reaction  PMMoV = Not measured | PMMoV dilution of 1/10. | None. | Monitoring drift, inhibition, and re-runs of sudden extreme peaks and falls of N1 and N2. | N1,  PMMoV multiplex, N2 as single plex.  No modification. |
| Toronto Metropolitan University – Central | N1: 2.3 copies/reaction  N2: 2.7 copies/reaction,  PMMoV: Not Measured | N1: 25.1 copies/reaction,  N2: 12.8 copies/reaction  PMMoV: Not Measured | PMMoV dilutions of 1/10. | RNA N1 and N2 standards were used so every standard curve relies on the reverse-transcription step, run EDX periodically, and use ddPCR to check standard quantity. | Control charts to monitor all standards including PMMoV, use standard curves to check for individual drifts in quality of standards, and check qPCR triplicates within 0.5 standard deviation. | Use ATCC standards, quantified by ddPCR and EDX,  Master curve updated in Spring 2023; use aggregated dataset downloaded from the hub after July 1, 2023. |
| University of Toronto - Central | N1: 1.0 copy/reaction  N2: 1.0 copy/reaction  PMMoV: 1.0 copy/reaction | N1: 4.0 copies/reaction  N2: 4.0 copies/reaction  PMMoV: 16.0 (could be lower but never tried) | PMMoV dilutions of 1/10; inhibition called if the ratio of quantification is smaller than 5 (i.e., equivalent to 1CT difference) | Nanodrop to check concentration, 260/230 and 260/280 ratio | R^2^ above 0.988, efficiency between 95%-105%; variations in standard curves y-intercept (typically 0.5 CT), slope, two positive controls (typically 30% difference in quantification) in each assay against the historical data; standard deviation of the CT of the technical replicates smaller than 0.5 for quantification higher than 16 copy/reaction; For quantification, if the reactions show no amplification (N/A or N/D) or trace amplification (<0,5 copy/reaction), 0.5 copy/reaction is used in substitution. | None. |
| University of Waterloo - Central | N1: 3.0 copies/reaction  N2: 3.0 copies/reaction  PMMoV: Not Measured | N1: 7.0 copies/reaction  N2: 7.0 copies/reaction  PMMoV: Not Measured | MS2 spike-in concentration check. | Total RNA concentrations using a nanodrop, Control charting for shifts in standard curve y-intercepts, standard curve Cqs, positive control Cqs, and SQs (for all targets including inhibition spike-in). Standard deviation between replicates < 0.35, no amplification within 5 Cqs of samples or standards in the NTCs, NRTs, or extraction blanks. | Remove PMMoV outliers (±3 standard deviations). Determine abnormally high or low SARS-CoV-2 values and communicate uncertainty if needed. Monitor N1/N2 drift. | Use EDX as a standard for N1, N2, and N200 until June 28^th^, 2023, after which, the University of Toronto concatenated plasmid was used. Used an IDT gBlock gene fragment of PMMoV as the PMMoV standard until June 28^th^, 2023, after which the University of Toronto concatenated plasmid was used. |
| University of Guelph - Western | N1: 1.3 copies/reaction  N2: 1.3 copies/reaction  PMMoV: 1.3 copies/reaction | N1: 4.5 copies/reaction  N2: 4.0 copies/reaction  PMMoV: 2.9 copies/reaction | EDX SARS-CoV-2 RNA standard spike-in concentration check. | Measure RNA concentration with Qubit | Composite curves with 95% confidence intervals, inhibition check, standard curves run with each plate, re-runs of tech reps with >1ct variability, NTCs | IDT plasmid used before Omicron BA.1, U of T concatenated plasmid used after. PMMoV quantification changed as well. |
| McMaster University - Western | N1: 2.1 copies/reaction  E: 4.6 copies per reaction  MS2: 5.9 copies/reaction  PMMoV: 5.9 copies/reaction | N1: 8.7 copies/reaction  E: 17.3 copies/reaction  MS2: 252.0 copies/reaction  PMMoV: 650.0 copies/reaction | PMMoV dilution of 1/10. | MS2 spike-in concentration check. | We assess the standard curve for R^2 and efficiency, check for inhibition using a 1 in 10 dilution, look for outliers between technical replicate wells, and monitor for similar trends between the E and N1 targets over time. | None. |
| Western University - Western | N1: 1.0 copy/reaction  N2: 1.0 copy/reaction  PMMoV: 1.0 copy/reaction | N1: 3.0 copies/reaction  N2: 3.0 copies/reaction  PMMoV: ~30.0 copies/reaction | PMMoV dilutions of 1/5 and 1/50. | Each biological replicate is run in analytical triplicate. Each qPCR plate includes negative, no template controls. | Inhibition using diluted sample results and checks for measurements below LOD. If two technical replicates are zero, a zero will be reported. | Use the University of Toronto concatenated plasmid for standards. Efficiency between 90 and 110% and R^2^ value above 0.98 |
| University of Windsor - Western | N1: 5.0 copies/reaction  N2: 5.0 copies/reaction  PMMoV: 5.0 copies/reaction | N1: 17.3 copies/well  N2: 14.3 copies/well  PMMoV = 94.3 copies/well | VetMAX XENO Internal Positive Control RNA spike-in concentration check. | Measured RNA concentration with Nanodrop. | Standard curves y-intercept, slope, inhibition, contamination. Above LOD, if Ct values difference >1.5 in 3 replicates, repeat qPCR. | None. |
